# The novel chloroplast glucose transporter pGlcT2 affects adaptation to extended light periods

**DOI:** 10.1016/j.jbc.2023.104741

**Published:** 2023-04-23

**Authors:** Marzieh Valifard, Alisdair R. Fernie, Anastasia Kitashova, Thomas Nägele, Rebekka Schröder, Melissa Meinert, Benjamin Pommerrenig, Denise Mehner-Breitfeld, Claus-Peter Witte, Thomas Brüser, Isabel Keller, H. Ekkehard Neuhaus

**Affiliations:** 1 Plant Physiology, University of Kaiserslautern, Kaiserslautern, Germany; 2 Central Metabolism, Max Planck Institute of Molecular Plant Physiology, Potsdam, Germany; 3 Ludwig Maximilians University Munich, Faculty of Biology, Plant Evolutionary Cell Biology, Planegg-Martinsried, Germany; 4 Leibniz University Hannover, Molecular Nutrition and Biochemistry of Plants, Hannover, Germany; 5 Leibniz University Hanover, Institute of Microbiology, Hannover, Germany

**Keywords:** *Arabidopsis thaliana*, chloroplast, membrane protein, glucose transport, photosynthesis

## Abstract

Intracellular sugar compartmentation is critical in plant development and acclimation to challenging environmental conditions. Sugar transport proteins are present in plasma membranes and in membranes of organelles such as vacuoles, the Golgi apparatus, and plastids. However, there may exist other transport proteins with uncharacterized roles in sugar compartmentation. Here we report one such novel transporter of the Monosaccharide Transporter Family, the closest phylogenetic homolog of which is the chloroplast-localized glucose transporter pGlcT and that we therefore term plastidic glucose transporter 2 (pGlcT2). We show, using gene-complemented glucose uptake deficiency of an *Escherichia coli* ptsG/manXYZ mutant strain and biochemical characterization, that this protein specifically facilitates glucose transport, whereas other sugars do not serve as substrates. In addition, we demonstrate pGlcT2-GFP localized to the chloroplast envelope and that pGlcT2 is mainly produced in seedlings and in the rosette center of mature Arabidopsis plants. Therefore, in conjunction with molecular and metabolic data, we propose pGlcT2 acts as a glucose importer that can limit cytosolic glucose availability in developing pGlcT2-overexpressing seedlings. Finally, we show both overexpression and deletion of pGlcT2 resulted in impaired growth efficiency under long day and continuous light conditions, suggesting pGlcT2 contributes to a release of glucose derived from starch mobilization late in the light phase. Together, these data indicate the facilitator pGlcT2 changes the direction in which it transports glucose during plant development and suggest the activity of pGlcT2 must be controlled spatially and temporarily in order to prevent developmental defects during adaptation to periods of extended light.

Among all primary metabolites, sugars fulfill a remarkably broad function. This is because they are required both for energy metabolism and to fuel a wide number of anabolic reactions. Moreover, sugars serve as precursors for storage polymer biosynthesis, they are involved in controlling organ development and harvest yield, and they contribute to plant tolerance against biotic or abiotic stress stimuli (1, 2, 3, 4, 5, 6). Accordingly, it is unsurprising that intracellular sugar levels are sensed, and corresponding molecular information governs gene expression or influences post-translational protein modification, leading to adjusted systemic reactions (7, 8, 9, 10). In leaves, *de novo* sugar synthesis occurs *via* two pathways. First, during photosynthesis chloroplast-derived triose phosphates are exported into the cytosol and provide carbon precursors for sucrose biosynthesis (11). Second, during starch mobilization glucose and maltose are released in the stroma and after export into the cytosol also support sucrose biosynthesis (12).

Sugar transport across the plasma membrane is generally mediated by a large number of individual transporters belonging to three protein families, namely, sucrose and monosaccharide transporting Sugar Transport Proteins (STP), monosaccharide-specific carriers of the Monosaccharide Transporter (MST) family, and sucrose- and monosaccharide transporting **S**ugar Will Eventually Be Transported (SWEET) type carriers (13, 14, 15). As shown for Arabidopsis and other species, these three transporter families comprise about 80 individual isoforms in corresponding genomes (3, 14).

Besides sugar transport across the tonoplast and plasma membrane, sugar transport has also been demonstrated across the plastid inner-envelope membrane (16, 17, 18, 19). Chloroplasts bear remarkable properties in that they harbor several metabolic pathways critical for plants and because they additionally represent molecular hubs required to orchestrate plant acclimation to various stress stimuli (20, 21, 22). Given such tight cellular integration, it seems worth to mention that so far only three sugar transporters have been identified in the plastid envelope on the molecular level. Those are the plastidic Glucose Transporter (pGlcT) (23), the Maltose EXporter1 (MEX1) (24), and the recently discovered plastidic Sugar Transporter (pSuT) (25). The latter transporter catalyzes sucrose export out of chloroplasts prior to onset of flowering or during cold acclimation (25), whereas for pGlcT and MEX1 a sugar export function during starch mobilization has been claimed. Although *pGlcT* loss-of-function mutants do not exhibit alterations of starch homeostasis or other phenotypic peculiarities, observations on double mutants lacking pGlcT and MEX1 are interpreted as indicative for a function of pGlcT in the export of glucose during starch degradation (26). This assumption is further reinforced by the analysis of the expression profile of the *pGlcT* gene in various rice tissues (27). In contrast, the marked accumulation of maltose and the starch excess phenotype of MEX1 knockout plants, as well as the specific expression of the *MEX1* gene in starch mobilizing plant tissues, unequivocally support the notion that MEX1 exports maltose during starch mobilization in the dark (24, 28).

A detailed phylogenetic analysis of the MST-type sugar transporter family in Arabidopsis revealed that pGlcT (encoded by the gene *At5g16150*) is one of four closely related carrier isoforms representing an independent MST subgroup (3). The next homolog to pGlcT is the protein encoded by the gene *At1g05030*. In contrast to pGlcT, no report on the protein encoded by the gene *At1g05030*, hereafter referred to as pGlcT2, is available. However, an in-depth characterization of pGlcT2 properties and its putative impact on Arabidopsis properties is mandatory for various reasons. First, neither the biochemical features of pGlcT2 nor its subcellular location or its gene expression pattern have so far been elucidated. Second, it is well known that the regulation of the cytosolic sugar concentrations can be of marked importance for yield and stress tolerance, and organelles including chloroplasts contribute to this regulatory process (25, 29, 30, 31). Thus, an analysis of the corresponding transporter function is important to complete our understanding of plant sugar homeostasis.

To this end, we initiated a comprehensive effort to widen our knowledge on this membrane protein. In the course of that work, we developed a recombinant *Escherichia coli*–based transport system allowing us to characterize the biochemical transport properties of pGlcT2. Further molecular analysis of the expression of *pGlcT2* and detailed characterization of the properties of knock-out and overexpressing mutants under various conditions showed that the activity of pGlcT2 must be controlled by Arabidopsis to prevent negative developmental/adaptational effects.

## Results

### The sequence of pGlcT2 exhibits canonical features of a chloroplast-located sugar transport protein and is widely present in vascular plants

pGlcT is one of 53 members of the MST family present in Arabidopsis (32). This MST family is clustered in 7 individual groups, and pGlcT belongs to a small subgroup comprising only four members (3). The structurally closest homolog to pGlcT is the putative carrier encoded by the gene *At1g05030*, which we named pGlcT2 (Fig. S1).

The molecular architecture of pGlcT2 demonstrates that this protein is comprised of 524 amino acids and exhibits 12 predicted transmembrane domains (TM) (Fig. S1). Accordingly, pGlcT2 shows the *bona fide* size and structure of typical sugar transport proteins belonging to the Major Facilitator Superfamily (MFS), present in bacteria and eukaryotes (33). The occurrence of conserved peptide sequences in pGlcT2 that are typical for sugar porters (34) further supports the assumption that this protein is able to transport sugar. These conserved patterns are the sequences -GXXLFGY- in TM1, -DxxGRR- between TMs 2/3 and 8/9, respectively, -PxSPRWL-between TMs 6/7, -VLYYXX-in TM7, and -VPETKG-that locates C-terminally adjacent to TM12 (Fig. S1). All these motifs are conserved among Major Facilitator Superfamily (MSF) -type sugar transporters as is the number of transmembrane domains (35).The pGlcT2 exhibits an N-terminally located amino acid extension that lacks any sequence domains conserved in sugar porters (34) (Fig. S1). That this sequence extension serves as a plastidic transit peptide is indicated by the following observations: (i) the chloroplast sugar transporter pSuT is the closest homolog to the vacuolar glucose transporter VGT1 (25). However, only the chloroplast-located protein pSuT exhibits an N-terminal located sequence extension similar to the corresponding structure in pGlcT2. (ii) in addition, the occurrence of a large number of serine residues in this pGlcT2 sequence extension (Fig. S1) suggests that this domain acts as a transit peptide required to post-translationally target the protein into the inner envelope membrane of chloroplasts (36).

As expected for plastidal transit peptides, the N-terminal 10 positions lack charged residues, serine and threonine residues are highly enriched (16% and 12%, respectively), the central domain lacks negative charges (in fact the whole transit peptide lacks negative charges), and the C-terminal 10 residues are rich in arginines and lysines (Fig. S1) (36). Such transit peptides do not share significant sequence conservation and are not believed to form folded domains other than possibly an amphiphilic β-strand that includes the positive charges near the C-terminal cleavage site (36). Consequently, the N-terminal extension of pGlcT2 is predicted by TargetP-2.0 to be a plastid-targeting transit peptide (37, 38).

From 28 plant species covering a wide phylogenetic range, members of the pGlcT/pGlcT2 MST subfamily were analyzed by phylogenetic clustering (Fig. S2). pGlcT2 proteins form a phylogenetic group that is well separated from the pGlcT cluster indicating that pGlcT2 has a distinct functional role, which is conserved in mosses and vascular plants. Although pGlcT2 is very widespread in plants, it is missing in several species of the Brassicaceae and also in duckweed (*Spirodela polyrhiza*) and seegrass (*Zostera marina*), indicating that pGlcT2 is dispensable in certain species either because its function may not be required there or may have been taken over by another protein.

### pGlcT2 is a glucose transporter

Prior to any in-depth characterization of pGlcT2, we examined whether this transporter harbors the ability to transport glucose. For this purpose, we chose a bacterial complementation system. In *E. coli*, glucose import is achieved by two transport systems, the glucose-specific phosphotransferase system transporter PtsG and the transporter ManXYZ, which efficiently transports a wider range of sugars, including mannose, glucose, fructose, mannosamine, glucosamine, or 2-deoxyglucose (39). We hypothesized that if pGlcT2 would function as a glucose transporter, it should permit the growth of an *E. coli ptsG/manXYZ* mutant strain on a minimal medium with glucose as the sole carbon and energy source.

For such analysis, it is mandatory to express the functional pGlcT2 in *E. coli*. To achieve constitutive gene expression, and to avoid protein abundances that could harm the cells or result in inclusion body formation. For this purpose, we chose the P_*TatA*_ promoter of *E. coli* in combination with a p15 origin vector, which has been shown to constitutively produce about 50-fold increased levels of membrane proteins (36). Two expression vectors were constructed, one (pABS-*pGlcT2a*-H6) for the production of the full-length protein with its N-terminal transit peptide for targeting across the plastid envelope membranes and another (pABS-*pGlcT2b*-H6) for production of the “mature” transporter without the transit peptide (see Experimental procedures). The removal of the transit peptide was expected to more likely result in complementation as the transit peptide is not required in the bacterial system and as bacteria do not possess transit peptide peptidases. Thus, the N-terminal extension is likely to interfere with membrane targeting and/or folding. As a negative control for the complementation analysis, we used the vector pABS-*tatC* for the production of TatC, a membrane protein that is involved in protein translocation and unrelated to sugar transport (40). These plasmids were transformed into *E. coli* MG1655 Δ*ptsG*/Δ*manXYZ* (41), and the resulting strains were precultured on M9 minimal medium with 0.4% xylose, before being transferred to M9 minimal medium with 0.4% glucose as the sole carbon and energy source.

**Figure 1:**
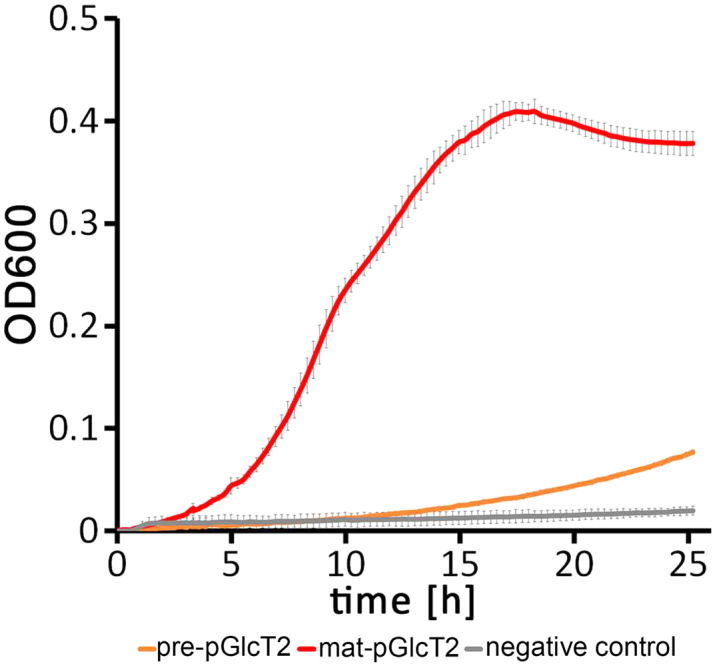
**pGlcT2 complements glucose uptake deficiency in *Escherichia coli ΔptsG ΔmanXYZ* mutants.** Growth of *E. coli* MG1655 *ΔptsG ΔmanXYZ* containing either pABS-*pGlcT2a*-H6 (pre-GlcT2, *brown line*), pABS-*pGlcT2b*-H6 (mat-GlcT2, *red line*), or pABS-*tatC* (negative control, *grey line*) in M9 medium with 0.4% glucose as only carbon and energy source. Error bars indicate the SD of technical triplicates. Note that the OD600 was recorded by a 96-well multi-titer plate reader with a 5.3 mm initial path length. pGlc2, plastidic glucose transporter 2.

The strain containing pABS-*pGlcT2b*-H6 grew well on this medium, indicating that pGlcT2 complemented glucose import into the bacterial cytoplasm when produced without the transit peptide for plastid targeting (Fig. 1, red curve). The strain containing pABS-*pGlcT2a*-H6 showed a very delayed and slow growth, which still exceeded that of the negative control, indicating that some functional pGlcT2 was also formed in this strain although the presence of the transit peptide clearly reduced the functionality of the transporter when produced in *E. coli* (Fig. 1, brown curve). As expected, the strain containing pABS-*tatC* did not grow on M9/glucose minimal medium, demonstrating that the *ptsG/manXYZ* deletion strain per se was not able to grow on glucose as a carbon source. Together, these data indicated that pGlcT2 can transport glucose.

### pGlcT2 transport is restricted to glucose transport

**Figure 2:**
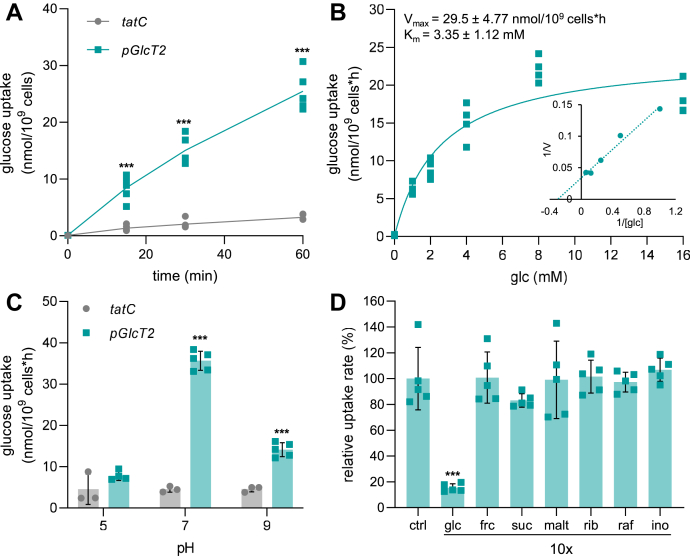
**Transport activity of pGlcT2 in *Escherichia coli ΔptsG ΔmanXYZ* mutants**. *A*, time-dependent glucose uptake activity was measured in *E. coli MG1655 ΔptsG ΔmanXYZ* expressing *pGlcT2* or *tatC*, serving as a negative control. *E. coli* cells were incubated with 5 mM of total glucose concentration containing 1 μCi of [^14^C] glucose at pH 7.0 for the indicated time intervals. Results represent means ±SD (n = 5). Significant differences in glucose uptake were calculated between cells expressing *pGlcT2* or *tatC*. *B*, substrate dependency of glucose import in *E. coli* expressing *pGlcT2*. Uptake activity was determined for different concentrations of ^14^C-glucose at pH 7.0 and calculated as difference between import in cells expressing *pGlcT2* or *tatC*. Results represent means ±SD (n = 4). *C*, pH dependency of glucose import in *E. coli* expressing *pGlcT2*. Import activity was determined at pH 5.0, 7.0 and 9.0 for *E. coli* expressing *pGlcT2* or *tatC* at a given ^14^C-glucose concentration of 5 mM. Results are means ±SD (n = 3 for *tatC*, n = 4–5 for *pGlcT2*). Significant differences were calculated between uptake activity in cells expressing *pGlcT2* and *tatC*. *D*, analysis of substrate specificity of *pGlcT2* as expressed in *E. coli*. Binding capacity of pGlcT2 for different sugars (glc: glucose; frc: fructose; suc: sucrose; mal: maltose; rib: ribose; raf: raffinose; ino: inositol) was determined by competitive inhibition of ^14^C-glucose uptake (2 mM initial outside concentration) in the presence of non-radioactive sugars in ten-fold excess at pH 7.0. Results are means ±SD (n = 5). Significant differences were calculated between the uptake activity in the control and the corresponding test conditions. Significance was calculated using Student's *t* test with ∗∗: *p* <0.01; ∗∗∗: *p*< 0.001 in all cases. pGlc2, plastidic glucose transporter 2.

To identify the transport properties of pGlcT2, we analyzed the import of radioactively labeled glucose into the functional *E. coli* complementation strain containing pABS-*pGlcT2b*-H6 (Fig. 2). At pH 7.0, the import was linear for 60 min, while the *tatC*-expressing control cells hardly accumulated radioactivity (Fig. 2*A*). A substrate saturation experiment revealed a V_max_ for glucose uptake of 29.5 nmol/10^9^ cells/h and an apparent affinity (K_m_) of 3.35 mM (Fig. 2*B*). This K_m_ suffices to explain the growth-supporting uptake in the *E. coli* system, which used a medium containing 0.4% (ca. 22 mM) glucose.

At an external pH of 5.0, ^14^C-glucose uptake in both, *pGlcT2* and *tatC*-expressing *E. coli* was similarly low. At pH 7.0, ^14^C-glucose uptake rose strongly in *pGlcT2*-expressing cells while it remained low in control cells expressing *tatC* (Fig. 2*C*). At pH 9.0, the rate of ^14^C-glucose uptake was significantly lower when compared to the uptake at pH 7.0 (Fig. 2*C*).

To raise our knowledge on the substrate spectrum of pGlcT2, we conducted competition experiments. For this, we incubated *pGlcT2-*expressing *E. coli* cells in ^14^C-glucose (at 2 mM as a control rate of uptake) and analyzed the effect of the simultaneous presence of putatively competing substrates (each at 20 mM). From all metabolites tested, solely non-labeled glucose decreased the rate of ^14^C-glucose uptake, while fructose, sucrose, maltose, ribose, raffinose or the sugar alcohol inositol did not affect the import of ^14^C-glucose (Fig. 2*D*).

### pGlcT2 locates in the chloroplast inner envelope

**Figure 3:**
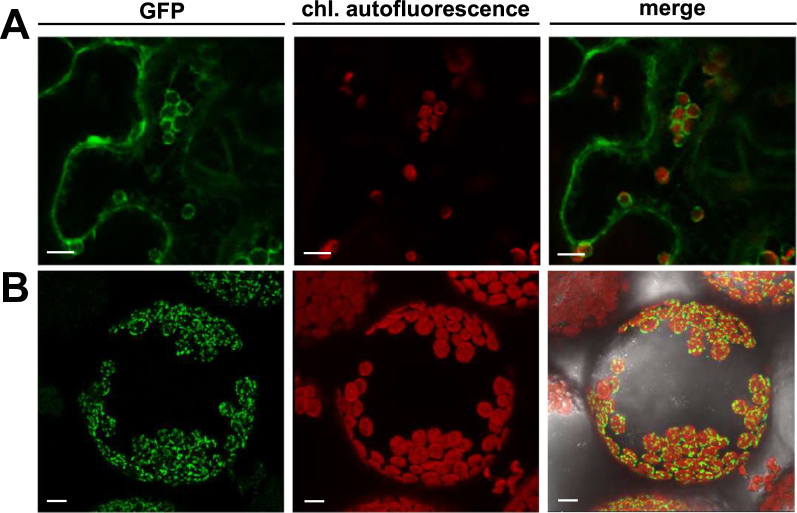
**Subcellular localization of pGlcT2-GFP.** Transient expression of the pGlcT2-GFP constructs in leaf mesophyll cells of *Nicotiana benthamiana*. The GFP fluorescence largely colocalized to the chlorophyll autofluorescence which demonstrated a chloroplast localization for the pGlcT2 protein. Confocal images of the GFP-signal, chlorophyll autofluorescence, and the merged view of both in transiently transformed tobacco mesophyll cells (*A*) and protoplasts (*B*) producing pGlcT2-GFP. Images were taken using a Leica TCS SP5II confocal laser scanning microscope. Scale bars represent 10 μm. pGlc2, plastidic glucose transporter 2.

Given the above findings, the biochemical and physiological characterization of pGlcT2 in plants became the focus of the remainder of this study. To examine the predicted subcellular localization of pGlcT2, we expressed a *pGlcT2-GFP* fusion transiently in intact tobacco mesophyll cells and isolated tobacco protoplasts (Fig. 3). The GFP signal surrounded the red (auto)fluorescence of the tobacco chloroplasts, and this fluorescence was completely absent in other cellular structures (Fig. 3, *A* and *B*). As such, these data experimentally confirmed the assumed chloroplast location of this transporter.

### The *pGlcT2* gene is expressed in young leaves and flower tissues

To analyze the expression of *pGlcT2* in Arabidopsis, we generated corresponding promotor-GUS mutants. To this end, we fused a 1992-bp fragment upstream of the start-ATG of *pGlcT2* to the gene *uidA*, coding for the ß-glucuronidase (GUS) reporter enzyme. This construct was used for the transformation of *Arabidopsis thaliana* Col-*0* plants.

**Figure 4:**
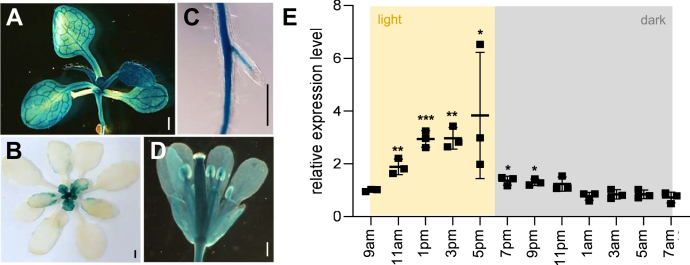
**Expression pattern of Pro-pGlcT2:GUS.** Histochemical localization of Pro-pGlcT2:b- GUS activity in 12-day-old seedling (*A*), 5-week-old rosette (*B*), lateral root (*C*), and single flower (*D*). Scale bars represent 2 mm in (*A*, *B* and *D*) and 1 mm in (i). *E*, diurnal expression of *pGlcT2* in wild-type plants grown under standard conditions for 4 weeks. Results are means of three biological replicates ± SD. Significant differences were calculated between the expression level at 9 AM and the corresponding *pGlcT2* expression throughout the day using Student's *t* test with ∗: *P* < 0.05; ∗∗: *P* <0.01; ∗∗∗: *P*< 0.001. GUS, GLUCURONIDASE; pGlc2, plastidic glucose transporter 2.

Representative pictures demonstrate that the *pGlcT2* in developing plants is mainly expressed in the leaf vasculature and less intense in mesophyll cells (Fig. 4*A*). In rosettes from adult plants, *pGlcT2* expression is mainly present in young leaves in the rosette center (Fig. 4*B*). Besides this, we observed a substantial expression of *pGlcT2* in the central cylinder of the primary root, in young lateral roots (Fig. 4*C*), and strong *pGlcT2* expression was seen in flower tissues like petals and anthers (Fig. 4*D*).

Because pGlcT2 is a close homolog to pGlcT, a chloroplast-located transporter with a function in starch degradation (23, 26), diurnal effects on *pGlcT2* expression were analyzed. For this, we harvested rosettes from plants grown under short-day conditions (10 h light) at various time points, extracted the mRNA, and quantified *pGlcT2* expression *via* qRT-PCR (Fig. 4*E*). It turned out, that the *pGlcT2* expression is induced after the onset of the light phase and the highest levels of mRNA accumulation were reached in the late light phase (Fig. 4*E*). Already within the first hour of darkness the *pGlcT2* mRNA level dropped to about 29% of the maximal level and plateaued at about 18% of the maximal level toward the end of the night phase (Fig. 4*E*).

### Identification of a pGlcT2 loss-of-function mutant, overexpression lines, and a complementation

To uncover the physiological role of pGlcT2 in more detail, we identified an Arabidopsis T-DNA insertion line lacking intact *pGlcT2* mRNA. The Salk_052078 line carries a T-DNA insertion in the first intron of *pGlcT2* (Fig. S3*A*). Since semi-quantitative RT-PCR analyses revealed an almost complete absence of *pGlcT2* in this line (Fig. S3*B*), it is considered as a loss-of-function (knock-out) mutant, named '*glct2'*.

To study the effects of increased pGlcT2 activity, we created, on the basis of the *glct2* line (Fig. S3*B*), two independent overexpression lines in which the *pGlcT2* cDNA was set under the control of the 35S cauliflower mosaic virus promotor (35S-CaMV). We identified in total 10 independent mutant lines in which the *pGlcT2* mRNA levels ranged from wild-type levels to up to 14-fold more *pGlcT2* mRNA (compared to wild-type). We chose line *glct2-comp* as a complemented plant line since it exhibited *pGlcT2* mRNA levels nearly identical to wild-type levels, and *pGlcT2* lines #8 and #5 as overexpression lines with 4- and 14-fold increased *pGlcT2* mRNA levels, respectively (Fig. S3, *D* and *E*).

### Germinating *pGlcT2* mutant plants exhibit altered development and modified carbohydrate levels

Having observed that *pGlcT2* is highly expressed in young Arabidopsis tissues (Fig. 4, *A* and *B*), it was of interest to check early plant development of corresponding mutant plants. To this end, we analyzed the germination pattern of wild types, *glct2* knockout plants, and the *pGlcT2*-overexpressing lines on MS agar medium, which either lacked additional sucrose or was supplemented with 0.5% sucrose, respectively. Sucrose effects were tested since it is known that the presence of this type of sugar allows homogeneous germination and promotes seedling development of *A. thaliana* and other species (42, 43, 44, 45).

**Figure 5:**
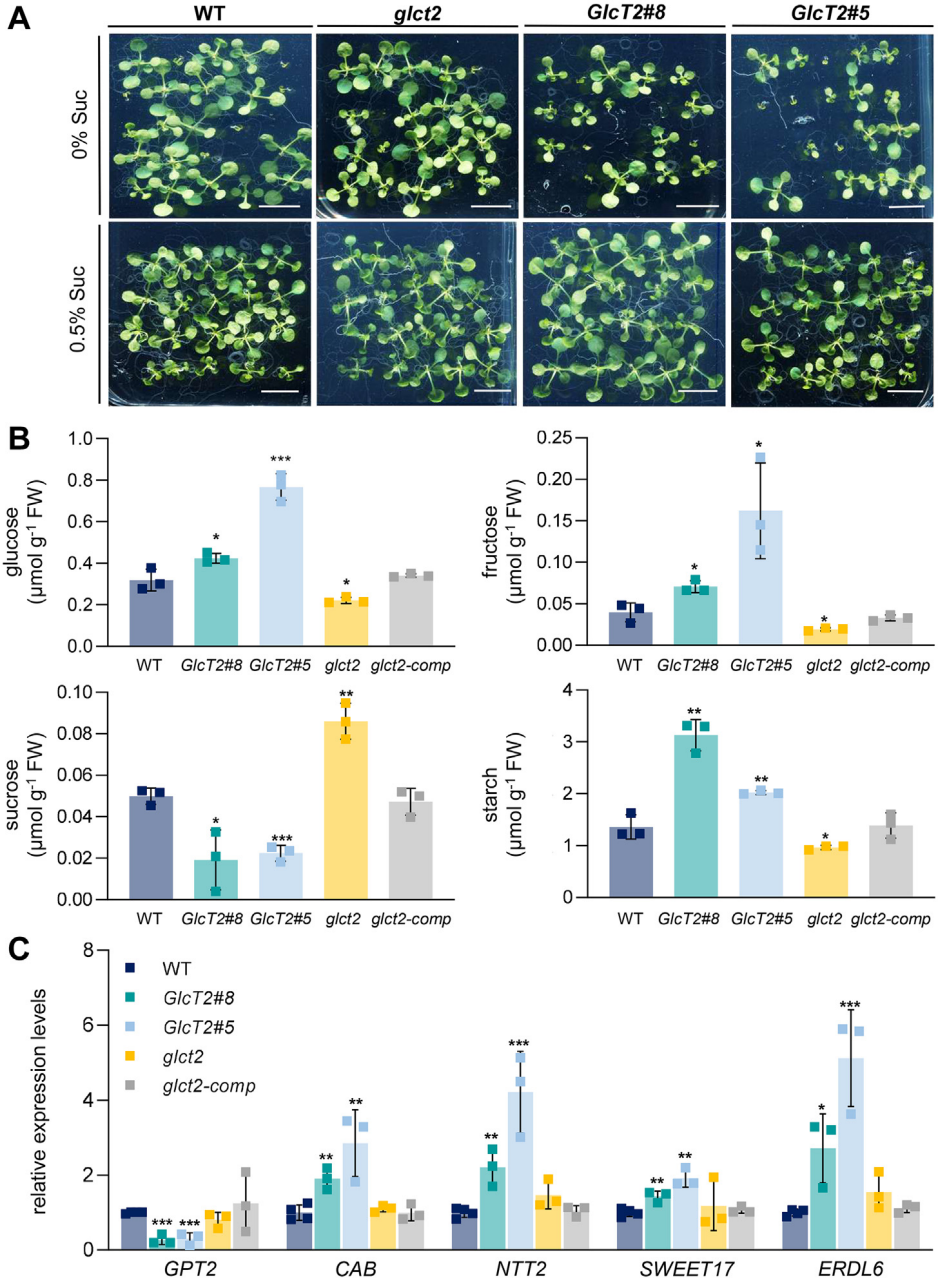
**Analysis of 2-week-old *Arabidopsis thaliana* pGlcT2 mutant seedlings on agar medium without external sugar supply.** The phenotype of 2-week-old WT, p*GlcT2* knockout, and overexpressor seedlings grown on MS-medium with 0% and 0.5% of sucrose (*A*), sugar and starch contents of 2-week-old seedlings grown on MS-medium with 0% sugars (*B*) as well as gene expression analysis of 2-week-old seedlings grown on MS-medium with 0% sugars (*C*). Seeds were sown on agar plates including 0 to 0.5% sucrose, kept at 4 °C/darkness for 48 h and transferred to short-day growth conditions and grown vertically for 2 weeks. Whole seedlings were harvested at midday, frozen in liquid nitrogen and remained at −80 °C until analysis. Scale bars represent 1 cm in (*A*). Results are means of three replications ± SD in (*B*) and means of 3 to 4 replications ± SD in (*C*). Each replicate consists of 80 to 100 seedlings. Significant differences were calculated between WT and corresponding mutants using Student's *t* test with ∗: *P* < 0.05; ∗∗: *P* <0.01; ∗∗∗: *P*< 0.001. pGlc2, plastidic glucose transporter 2.

The germination pattern of wild types on agar lacking sucrose is not uniform as a few less-developed seedlings appeared always among well-developed plants (Fig. 5*A*). A similar germination pattern was observed for the *glct2* mutant, while overexpression plants exhibited an impaired germination efficiency (Fig. 5*A*). In contrast, in the presence of 0.5% sucrose germination of seeds was highly homogeneous and no differences between mutants and wild types were visible (Fig. 5*A*).

Having seen that *pGlcT2* mutants exhibit a germination phenotype in the absence of sucrose (Fig. 5*A*) we were interested to quantify levels of carbohydrates in all plant lines when grown without an additional sugar source. Because the *glct2* knockout line exhibited a metabolic pattern different from wild types (see below), we included the complemented line in this metabolic analysis.

Two-week-old seedlings of the *pGlcT2* overexpression line #5 exhibit higher glucose and fructose levels than those present in the corresponding wild types. A similar tendency was also seen for *pGlcT2* overexpression line #8 (Fig. 5*B*), representing a weaker overexpression mutant (Fig. S3*E*). However, both *pGlcT2* overexpression lines exhibited lower sucrose levels when compared to wild types, ranging between 0.02 to 0.026 μmol g^−1^ FW, which is about half of the wild type level (0.05 μmol g^−1^ FW) (Fig. 5*B*). Interestingly, while sucrose levels are decreased in *pGlcT2* overexpressors when compared to wild types, *pGlcT2* overexpression line #8 exhibits nearly doubled levels of starch when compared to wild types, namely, 3 μmol *versus* 1.5 μmol C6 g^−1^ FW (Fig. 5*B*). When compared to wild types, *pGlcT2* overexpression line #5 exhibited in tendency slightly higher starch levels (Fig. 5*B*).

Remarkably, the carbohydrate pattern found in the knock-out line *glct2* is opposite to the carbohydrate pattern present in overexpressor plants. While both, glucose and fructose concentrations are decreased when compared to corresponding wild types, sucrose concentrations reached 0.08 μmol g^−1^ FW (Fig. 5*B*), representing a 1.7-fold increase. Starch, while increased in both overexpressor lines, appeared in a tendency to be decreased in the *glct2* knock-out line (Fig. 5*B*). Carbohydrate levels in the complemented line (*glct2-comp*) exhibited no differences when compared to levels in correspondingly grown wild types (Fig. 5*B*), indicating that the complementation of the knock-out line was successful.

It is well known that alterations in cellular sugar levels influence the expression of many nuclear-encoded genes involved (46, 47). Thus, we checked the expression of selected sugar-affected genes in wild types and the transgenic lines such as *GPT2*, a glucose-induced gene coding for the plastidic glucose 6-phosphate transporter 2 (48). The expression of *GPT2* is strongly downregulated in leaves from both overexpressor lines when compared to wild-type levels and to *GPT2* mRNA concentrations present in knockout plants, or the complemented line *glct2-comp* (Fig. 5*C*). The *NTT2* gene, coding for the chloroplast located ATP importer (49), is up to 4.5-fold higher expressed in *pGlcT2* overexpressor plants (Fig. 5*C*). The genes *SWEET17* and *ERDL4*, coding for two vacuolar sugar exporters, are up to 5-fold higher expressed in the two overexpressor lines when compared to wild types, but essentially unchanged in the *glct2* knock-out line, and almost identical to wild types in the complemented mutant line (Fig. 5*C*).

### *pGlcT2* mutants show impaired growth efficiency and exhibit altered carbohydrate homeostasis under extended day lengths

**Figure 6:**
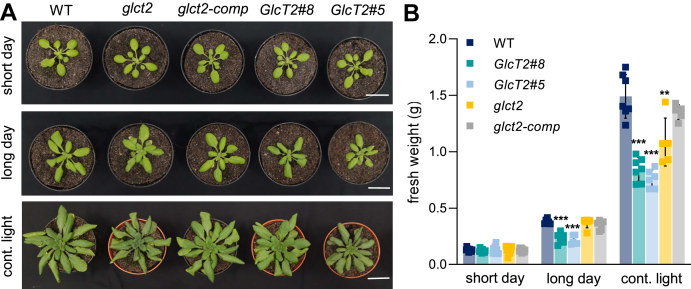
**Growth analysis of *Arabidopsis thaliana* pGlcT2 mutant lines under long day and continuous light conditions.** Plant phenotype (*A*) and plant biomass (*B*) under short day, long day, and continuous light. Seeds were sown on soil, stratified at 4 °C/darkness for 48 h and transferred to standard growth conditions (short day). After pricking, plants were either kept in short-day (control) or long-day (treatment) chambers for 24 days. To apply continuous light, plants that were grown under standard conditions for 24 days, were transferred to continuous light for another 10 to 12 days. All lines were harvested at midday per each growth condition. Scale bars represent 2 cm in (A). Results are means of 5 to 10 replicates ± SD. Significant differences were calculated between WT and corresponding mutants within one condition using Student's *t* test with ∗: *P* < 0.05; ∗∗: *P* <0.01; ∗∗∗: *P*< 0.001. pGlc2, plastidic glucose transporter 2.

Having seen that *pGlcT2* mutants exhibit a growth phenotype during early development (Fig. 5*A*) and knowing that *pGlcT2* gene expression is markedly stimulated during the late light phase (Fig. 4*E*) we analyzed the growth pattern under short-day and extended-day length conditions, *e.g.* long-day length (20h light) and continuous light (Fig. 6).

Under short-day conditions all five plant lines exhibited a nearly similar growth pattern, leading on average to a rosette biomass of about 0.12 g (Fig. 6, *A* and *B*). In contrast, under long-day conditions both *pGlcT2* overexpressor mutant lines showed impaired growth efficiency, leading to rosette biomasses of 0.22 g and 0.25 g, respectively, which represent about 75 to 70 % of the biomass exhibited by wild types (Fig. 6*A*). The growth patterns of both, the *glct2* knock-out line and the complemented line *glct2-comp* did not differ from the growth pattern of wild types (Fig. 6, *A* and *B*).

Interestingly, under continuous light conditions, the two *pGlcT2* overexpressors and the *glct2* knockout line exhibited impaired growth efficiencies. The overexpressors reached in average 0.76 g and 0.84 g rosette biomass, respectively, representing about 60 % of the biomass gained by wild types (Fig. 6*B*, note, for technical reasons independent short-day controls have been conducted for the analysis of plants grown under continuous light conditions, see Fig. S4 and legend to this). Similarly, the *glct2* knock-out line now also exhibited impaired growth efficiency and reached only 1.0 g rosette biomass, representing about 75 % of the biomass exhibited by wild types (Fig. 6*B*). The growth pattern of the complemented line *glct2-comp* did not differ significantly from wild types (Fig. 6*B*).

**Figure 7:**
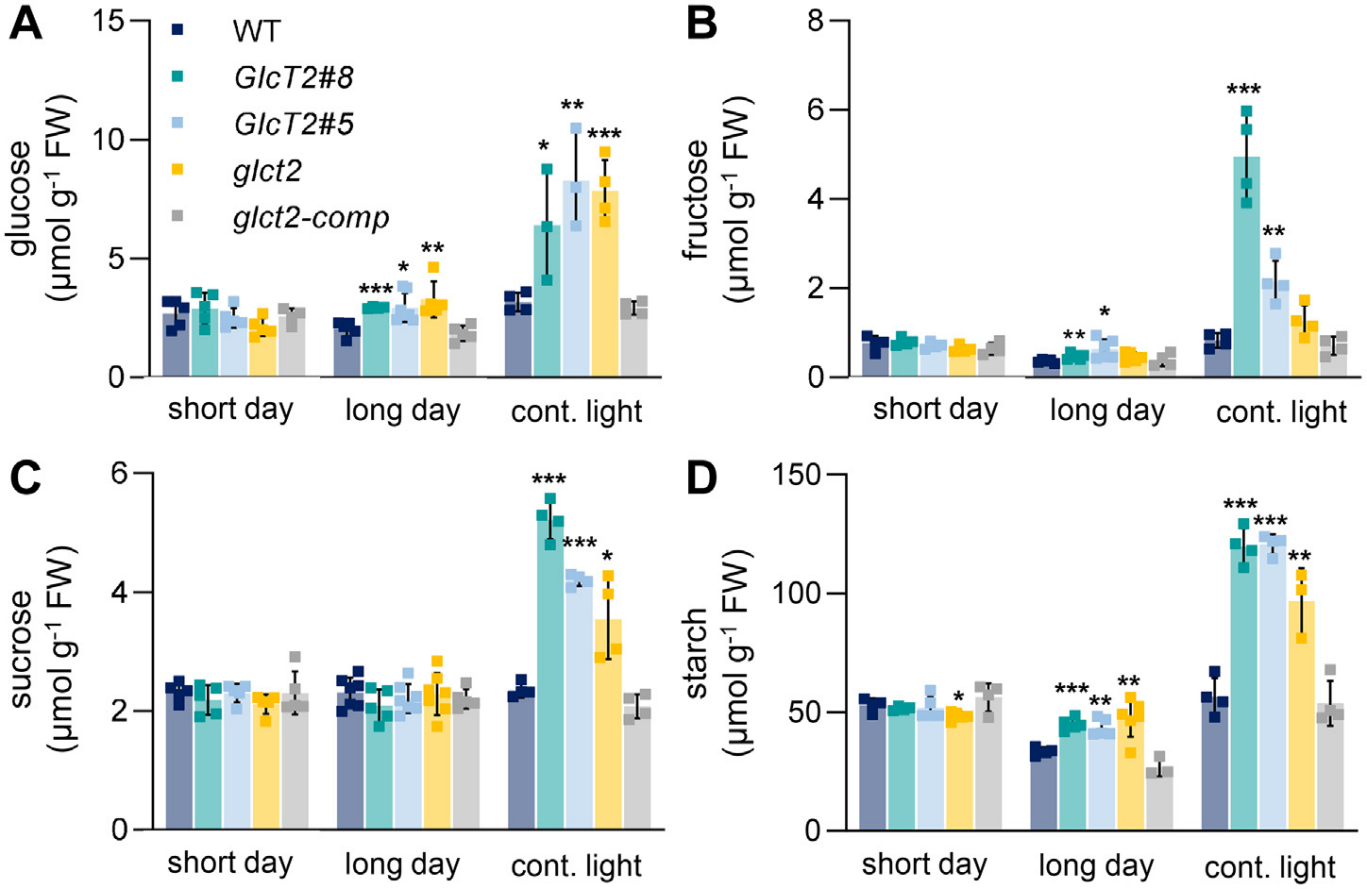
**Sugar and starch content of *Arabidopsis thaliana* pGlcT2 mutant plants grown under short-day, long-day, and continuous light.** Carbohydrate levels of 24-day-old plants grown under short day and long day conditions. For continuous light conditions, plants were initially grown under short-day conditions for 24 days and subsequently transferred to continuous light for another 10 to 12 days. Sugar levels are presented as μmol glucose (*A*), fructose (*B*), sucrose (*C*), and hydrolyzed starch (*D*) per Gramm fresh weight. Data are shown as mean ± SD of 3 to 7 biological replicates. Significant differences were calculated between WT and corresponding mutants within one condition using Student's *t* test with ∗: *P* < 0.05; ∗∗: *P* < 0.01; ∗∗∗: *P* < 0.001. pGlc2, plastidic glucose transporter 2.

As stated above, under selected conditions the mutant lines exhibited altered carbohydrate levels during early plant development (Fig. 5, *B* and *C*). To check also for altered carbohydrate levels under either short-day, long-day, or continuous light conditions, we quantified sugar and starch levels from wild types and mutant plants (please note: for technical reasons, we grew independent short-day controls for plants grown under continuous light conditions; for data see Fig. S4 and legend to this). When grown under short days, rosettes of all plant lines exhibited similar levels of glucose, fructose, sucrose, and starch (Fig. 7, *A*–*D*). In contrast, glucose levels in all three *pGlcT2* mutant types appeared to be increased when plants had been transferred to long-day conditions for the last 10 to 12 days of the growing period. The two *pGlcT2* overexpressor lines exhibited 2.9 μmol Glc g^−1^ FW, and the *glct2* knockout line contained 3.2 μmol Glc g^−1^ FW, which represents up to 30 % more glucose than present in wild types (Fig. 7*A*). This difference in the glucose levels is even more pronounced in mutants grown under continuous light (Fig. 7*A*). Under latter conditions, the two *pGlcT2* overexpressor lines #5 and #8 exhibited 6.4 and 8.3 μmol Glc g^−1^ FW, respectively, which represents up to 2.6-fold more glucose than present in wild types (Fig. 7*A*). The *glct2* knockout line contained 7.8 μmol Glc g^−1^ FW, which represents even 2.4-fold more Glc than present in wild types (Fig. 7*A*).

Under long-day conditions also fructose levels were increased in the two *pGlcT2* overexpressor lines, up to 38% when compared to wild-type levels, while unchanged in the knock-out mutant (Fig. 7*B*). In continuous light, fructose levels were increased in all three *pGlcT2* mutants, with the most marked changes in two *pGlcT2* overexpressor mutants. *E.g. pGlcT2* overexpressor line #8 contained about 4.5-fold higher fructose concentrations than present in wild types (Fig. 7*B*). Sucrose levels in all individual lines were nearly identical when plants were grown under long-day conditions (Fig. 7*D*). In contrast, in continuous light, the *pGlcT2* overexpressor lines exhibited 2- and 1.6-fold higher sucrose than present in wild types, and the knockout line exhibited 1.3-fold higher sucrose when compared to the concentrations in corresponding wild-type plants (Fig. 7*D*).

Starch levels in *pGlcT2* mutants were increased under long-day conditions. All three mutants accumulated starch equivalent to about 48 to 52 μmol C6 g^−1^ FW, representing a slight increase of about 25% when compared to corresponding levels in wild types (Fig. 7*D*). Interestingly, under continuous light conditions, the two *pGlcT2* overexpressor mutants #5 and #8 contained very high starch levels, namely 121 and 120 μmol C6 g^−1^ FW, respectively, and in the knock-out line starch amounted to 97 μmol C6 g^−1^ FW (Fig. 7*D*).

### *pGlcT2* mutants exhibit altered expression of genes related to carbohydrate homeostasis under continuous light conditions

As stated above, *pGlcT2* mutants show the most marked alterations of their carbohydrate (sugars and starch) metabolism when cultivated under continuous light conditions (Fig. 7). Moreover, this growth regime provokes the strongest effects on the growth pattern of these mutant lines (Fig. 6). Thus, we checked under continuous light conditions for alterations of the expression of genes coding for enzymes involved in sugar and starch metabolism. To this end, we prepared mRNA from plants grown in continuous light and conducted an RNA seq analysis (GEO repository GSE223330) from which we extracted genes coding for proteins with relevance for carbohydrate metabolism (Table 1).

**Table 1:** Log2-fold changes of genes involved in sucrose and starch turnover in *pGlcT2* mutant plants in comparison to WT grown under continuous light

It became obvious, that the expression of genes coding for individual isoforms of the cytosolic located enzymes sucrose phosphate synthase (SPS) and the subsequently acting enzyme sucrose phosphate phosphatase (SPP) were upregulated in the *pGlcT2* overexpressor line #5, while the expression of these genes was throughout down-regulated in the knock-out line *glct2* (Table 1). Similarly, the genes coding for the enzymes plastidic phosphoglucomutase, ADP glucose pyrophosphorylase (small subunit), soluble starch synthase, isoamylase and starch branching enzyme (which are all involved in starch accumulation) were upregulated in *GlcT2#5*, while (with the exception of *SBE3*) down-regulated in the knock-out line *glct2* (Table 1). In addition, all tested genes coding for enzymes required for starch degradation, namely disproportionation enzymes 1 and 2, β-amylases 1 and 3, and α-amylases 1, 2, and 3 were upregulated in the *pGlcT2* overexpressor line #5, while, with the exception of *AMY1*, these genes were downregulated in the *glct2* line (Table 1). The gene coding for the chloroplast located glucose-6-phosphate transporter *GPT2* was upregulated in both mutant lines, although the degree of upregulation was markedly higher in the *pGlcT2* overexpressor line #5 (5.20 log2FC in *GlcT2#5*, Table 1). The expression of the gene coding for the maltose exporter1 (*MEX1*) was increased in the *pGlcT2* overexpressor line #5, while down-regulated in the knock-out line *glct2*. The genes coding for three further chloroplast-located transporters, namely, the triose phosphate translocator *TPT*, *pGlcT*,and *pSuT* were downregulated in both mutant lines when compared to the corresponding gene expression in wild types (Table 1). We would like to exclude that pGlcT might take over some function in the *pGlcT2* knock-out plant *gtl* because the *pGlcT* gene hardly responds to the absence of a functional pGlcT2 protein.

### *pGlcT2* overexpressors show impaired photosynthetic activity

Since the growth of *pGlcT2* mutants was impaired under continuous light conditions (Fig. 6) we were interested to compare the photosynthetic performance of all types of plants when grown under either short-day conditions or under continuous light, by use of PAM fluorescence analysis.

**Figure 8:**
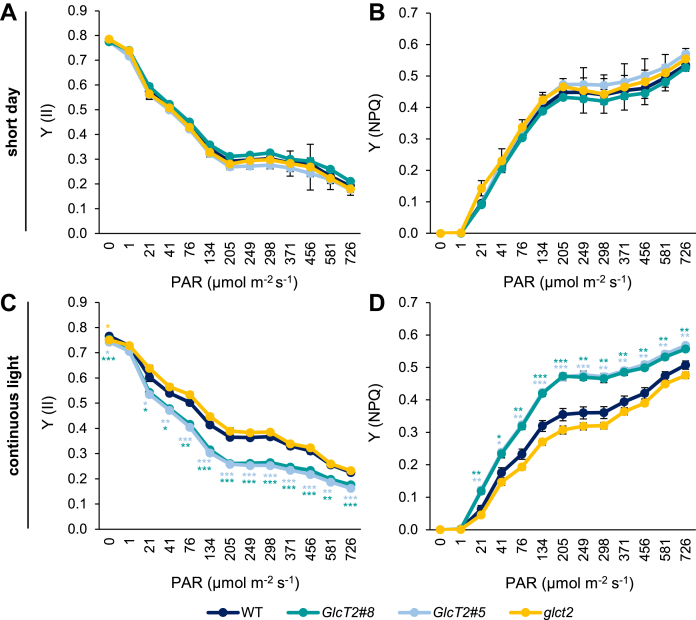
**Photosynthesis measurement of *Arabidopsis thaliana* pGlcT2 mutant plants grown under short day and continuous light.** Photosynthetic efficiency was evaluated on 3-week-old plants grown under short-day conditions (*A* and *B*) and continuous light (*C* and *D*). The effective quantum yield of PSII [Y(II)] (*A* and *C*) and the quantum yield of non-photochemical quenching [Y(NPQ)] (*B* and *D*) were determined using a light curve of increasing PAR intensity. Data are shown as mean ±SD of 5 biological replicates. Each biological replicate consists of 3 plants, which were grown and measured together in one pot. Significant differences were calculated between WT and corresponding mutants within one PAR using Student’s *t* test with ∗: *P* < 0.05; ∗∗: *P* < 0.01; ∗∗∗: *P* < 0.001. pGlc2, plastidic glucose transporter 2; WT, wild type.

When grown under short-day conditions, in which all plant types develop similarly (Fig. 6), the four lines analyzed exhibited nearly identical quantum yields (Y (II)) and non-photochemical quench (Y (NPQ)) properties (Fig. 8, *A* and *B*). In contrast, when growing under continuous light conditions, both *pGlcT2* overexpressor lines responded to rising light intensities with a lower quantum yield when compared to the corresponding responses of wild-type plants and the knock-out line (Fig. 8*C*). Similarly, rising light intensities induced markedly higher NPQ in both *pGlcT2* overexpressor lines than in wild types under identical conditions when grown in continuous light (Fig. 8*D*).

To identify putative reasons for the decreased photosynthetic performance of *pGlcT2* overexpressors, we reanalyzed our RNAseq data obtained from the overexpressor *pGlcT2#5* and the mutant strain *glct* when grown under continuous light conditions (Table 2). It turned out, that in both lines 20 out of 23 photosynthesis-relevant genes exhibited altered expression when compared to wild types. For example, the expression of genes coding for components of photosystem (PS) II and I was lowered in both lines (Table 2). However, the degree of down-regulation was markedly stronger in the *pGlcT2* overexpressor line #5 than in knock-out line *glct2* (Table 2). The four genes coding for the RuBisCO subunits A and B, namely *RbcS1A* and *RbcS1B*, *RbcS2B,* and *RbcS3B*, were downregulated in *GlcT2#5*. Again, the degree of down-regulation of gene expression for *RbcS1A* and *RbcS3B* was less pronounced in the *glct2* line, and the expression of *RbcS1B* and *RbcS2B* was, in contrast to the situation in the *pGlcT2* overexpressor line #5, slightly increased in the *glct2* plants (Table 2).

**Table 2:** Log2-fold changes of genes involved in photosynthesis in *pGlcT2* mutant plants in comparison to WT grown under continuous light

## Discussion

### pGlcT2 is a chloroplast-located sugar transporter and facilitates glucose transport

Up to now three carrier proteins able to transport neutral sugars across the chloroplast envelope have been identified on the molecular level, namely, the glucose-exporting protein pGlcT, the maltose exporter MEX1, and the sucrose exporting protein pSuT (23, 24, 25). While MEX1 exhibits a unique molecular architecture and occurs solely in the green plant lineage (24, 50, 51), both, pGlcT and pSuT belong to the large MSF of transporters, which mostly exhibit between 10 to 14 predicted transmembrane domains (52). Within the MSF, both pGlcT and pSuT cluster into the plant-specific MST family, comprising in total 53 members (3).

pGlcT2 represents a further sugar transporter of the inner chloroplast envelope, which is indicated by several lines of evidence. First, pGlcT2 exhibits the closest evolutionary relationship to the chloroplast sugar translocator pGlcT ((3) and Fig. S2). Secondly, pGlcT2 shows, similar to other sugar porters of the Major Facilitator Superfamily, 12 predicted transmembrane domains and contains several highly conserved domains (Fig. S1) representing canonical sugar binding structures (35). Thirdly, pGlcT2 possesses an N-terminal transit peptide for targeting nuclear-encoded proteins into the chloroplast (Fig. S1). This characteristic was initially predicted by TargetP-2.0 and could be experimentally confirmed, as a pGlcT2-GFP fusion protein was targeted to the chloroplast when transiently expressed in intact tobacco leaf cells or in protoplasts (Fig. 3). We would like to note that pGlcT2 has so far not been discovered in envelope proteome studies (see *e.g.* (37, 53, 54)). However, the absence of pGlcT2 in the latter studies does not contradict our conclusions, given that envelopes are routinely prepared from chloroplasts derived from mature Arabidopsis leaves, while *pGlcT2* is mainly expressed in young rosette leaves (Fig. 4*B*).

As given in Figure 4, *pGlcT2* gene expression is not limited to photosynthetically active tissues but is also detectable in heterotrophic tissues like roots or inflorescences. Accordingly, pGlcT2 is also present in non-green plastids which depend for anabolic reactions on the uptake of sugars from the cytosol. Such uptake is possible by *e.g.* the glucose-6-phosphate/Pi antiporter GPT2 (38) allowing import of Glc6P. However, it has also been shown that heterotrophic plastids are also able to import glucose (18, 55) and it remains to be analyzed to which degree pGlcT2 is involved in this process.

Interestingly, pGlcT2 is missing in several Brassica species and also in some relatively close relatives of *A. thaliana* like *Capsella rubella*, while generally it is found in almost all vascular plants and mosses (Fig. S2). The patchy conservation of pGlcT2 in Brassicaceae might indicate that, particularly in this group of plants, another transporter can easily acquire the capability to functionally compensate for a loss of pGlcT2.

Testing the function of a translocator is often hampered by the fact that the hydrophobic recombinant protein must be inserted into a membrane. To this end, we exploited a recombinant *E. coli* strain lacking most of its endogenous glucose transporters (41), and we used a constitutive intermediate-level expression system that has been established for functional membrane protein production to ensure proper membrane integration (36). This approach allowed us to reveal an exclusive glucose specificity of pGlcT2 (Fig. 2*D*). This specific substrate specificity differs from properties of the plastidic sugar transporter pSuT, which has been shown to accept both, the monosaccharide glucose and the disaccharide sucrose (25). In addition, whether this strict substrate specificity is a unique feature of pGlcT2 is unclear because for both, pGlcT and MEX1, a detailed biochemical analysis remains elusive (23, 24).

We observed that neither a low pH (pH 5.0) nor a high pH (pH 8.0) stimulates pGlcT2-mediated sugars transport above the rate observed at pH 7.0 (Fig. 2*C*). Thus, while the energetics of pGlcT and MEX1 are unclear our data indicate that pGlcT2 acts as a facilitator and is as this not driven by a proton-motive force. As a facilitator pGlcT2 differs in its energization to pSuT, which is a proton/sugar antiporter acting in the light phase as a sucrose exporter into the cytosol and contributing with this to the sucrose-induced flower initiation (25). In sum, we propose that pGlcT2 transports glucose along an existing substrate concentration gradient which, however, might change in dependence upon development or physiological conditions.

### Evidence that pGlcT2 acts as a glucose importer during early plant development

Given that pGlcT2 is a glucose facilitator (Fig. 2*C*) the direction of transport solely depends upon the relative glucose concentration on both sites of the inner chloroplast envelope membrane. To search for a putative effect of pGlcT2 under conditions where stromal glucose levels are low, we analyzed the properties of corresponding mutants during early plant development. During this phase of plant growth, the newly generated sucrose is hydrolyzed by the vacuolar invertase, as this enzyme is necessary to hydrolyze sucrose (Vu *et al*. 2020), previously derived from lipid mobilization and gluconeogenesis. The resulting monosaccharides are subsequently exported from the vacuole with the help of specific exporters, *e.g.* SWEET17 for fructose release and ERDL6 for glucose release (Chardon *et al*., 2013, Guo *et al*., 2013, Poschet *et al*. 2008) which in sum leads to sugar accumulation in the cytosol. In contrast to the cytosol, the hexose levels in the stroma from Arabidopsis are comparable low (56). Thus, any developmental peculiarity of Arabidopsis mutants exhibiting altered activity of the plastidial glucose facilitator pGlcT2 (Fig. 2*C*) must be discussed on the basis of this background information.

It is obvious that the two *pGlcT2* overexpressor lines exhibited impaired germination on MS agar plates when additional sucrose is not present, while in the presence of additional sugar, all plant lines develop similarly (Fig. 5*A*). This observation led us to propose that *pGlcT2* overexpressor mutants suffer from low sugar availability. However, such an assumption is challenged by the fact that under these selective conditions *pGlcT2* overexpressor mutants in fact exhibited increased levels of monosaccharides (Fig. 5*B*). Our corresponding explanation is that during germination without additional sugar source, pGlcT2 acts as a plastidial sugar importer transporting cytosolic glucose into developing chloroplasts. This hypothesis is fostered by the following observations: (i) *pGlcT2* overexpressor mutants contain less sucrose and (ii) *pGlcT2* overexpressor mutants contain more starch when compared to the levels in the corresponding wild types (Fig. 5*B*). It is known that during seedling development sucrose hydrolysis occurs in the Arabidopsis vacuole (57). Accordingly, an increased import of glucose into chloroplasts of *pGlcT2* overexpressor mutants would shift the reaction of the vacuolar invertase towards sucrose hydrolysis leading to decreased total sucrose levels (Fig. 5*B*). Concerning the increased starch levels in developing *pGlcT2* overexpressor mutants we assume that a fraction of the imported glucose is converted to glucose-6-phosphate (Glc6P) (which represents the *bona fide* precursor for starch biosynthesis) catalyzed by the plastidic hexokinase pHXK (58). This assumption is supported by the fact that *pGlcT2* overexpressor plants also exhibit increased expression of the *NTT2* gene, coding for the plastidic ATP importer (49, 59). It is known that the maturation of young chloroplasts depends upon the activity of NTT proteins (60) and obviously, the increased demand for ATP in the stroma, due to accelerated conversion of glucose into starch (Fig. 5*B*), is sensed, and induces the expression of *NTT2*, leading to a stimulation of ATP import into the developing chloroplast. It will be interesting to search for corresponding metabolic signals connecting altered plastidic ATP demand to *NTT2* expression.

Increased transport activity of pGlcT2 traps glucose in the developing chloroplasts and limits, therefore, carbon precursor availability in the cytosol and as a consequence also mitochondrial energy provision, leading in consequence to impaired early development of pGlcT2 overexpressor mutants (Fig. 5*A*). In fact, the assumption that cytosolic glucose levels are comparably low in the *pGlcT2* overexpressors gains independent experimental support by the fact that the expression of the glucose-repressed genes *CAB1*, *ERDL6*, and *SWEET17* (46, 61, 62) is higher in *pGlcT2* overexpressor seedlings when compared to wild types (Fig. 5*C*). The observation that *pGlcT2* loss-of-function mutants exhibit a similar gene expression pattern as observed in wild types (Fig. 5*C*) might be due to relatively small changes of the cytosolic sugar levels caused by the absence of pGlcT2 activity. This assumption finds supported by a recent report on the effects induced by altered maltose levels in Arabidopsis, where molecular responses were only detectable when the concentration of this sugar exceeds certain thresholds (63).

### *pGlcT2* mutants exhibit a peculiar phenotype with increasing day length which is due to impaired photosynthesis

When grown under short-day conditions, all plant lines exhibited similar rosette size (Fig. 6*A*). However, under long-day and throughout under continuous light conditions both, *pGlcT2* overexpressor mutants and knock-out plants (but not the complemented line) exhibited growth deficiencies (Fig. 6, *A* and *B*). This observation indicates that under specific light conditions, the activity of the pGlcT2 protein must be fine-tuned since an increased or insufficient activity negatively affects plant development.

Corresponding metabolic quantifications revealed that the impaired development of *pGlcT2* overexpressors under long day and more pronounced under continuous light led to increased levels of sugars and starch (Fig. 7, *A*–*D*). Interestingly, in particular, increased starch levels of *pGlcT2* overexpressors under extended light phases (Fig. 7*D*) resemble the metabolic profile observed in *pGlcT2* overexpressors during germination in the absence of sucrose (Fig. 5*B*). As stated earlier, we assume that pGlcT2 in overexpressors acts as a glucose importer during germination in the absence of sucrose. However, during long day conditions or in continuous light the markedly high starch levels in *pGlcT2* overexpressors are not due to increased glucose import, but rather due to increased glucose export into the cytosol.

For such a conclusion we must clarify both, the source of glucose in the stroma and the molecular consequences of increased cytosolic glucose in *pGlcT2* overexpressors when cultivated under extended light phases. The main source (or maybe even the sole source) of glucose in the chloroplast stroma is starch mobilization. This metabolic process is organized in a complex manner and one enzyme critically involved is the stromal-located disproportionating enzyme1 (DPE1) (64). DPE1, as a α-1,4-glucanotransferase (65), is responsible for the transfer of maltosyl units from one 1,4-α-d-glucan to another, leading to longer glycan structures which again serve as efficient substrates for phosphorylases and α-amylases (66). In addition to longer glycan structures, DPE1 releases during each reaction cycle one free glucose residue. Interestingly, independent reports demonstrate that chloroplasts start to mobilize starch in the late light phase, especially under conditions of extended day length (67, 68). Accordingly, in chloroplasts from Arabidopsis plants cultivated under long day- or under continuous light conditions the mobilization of starch leads to the stromal presence of glucose.

The assumption that this starch-derived glucose is rapidly exported into the cytosol of *pGlcT2* overexpressors, when compared to wild types, is supported by corresponding changes in the expression of glucose-affected genes. It is known that several genes coding for enzymes involved in starch synthesis are glucose-induced (46, 69), and the expression of 12 out of 13 genes coding for enzymes involved in starch synthesis is increased in the *pGlcT2* overexpressor line when compared to wild types (Table 1). It is worth mentioning that also selected genes coding for enzymes involved in starch degradation are glucose-induced (70). In fact, a slightly increased expression of genes coding for starch degradation enzymes can be observed in the *pGlcT2* overexpressor line (Table 1). The latter observation supports the assumption that glucose released from starch degradation in the light is the substrate for sugar export mediated by pGlcT2. Since genes coding for enzymes involved in sucrose biosynthesis are also higher expressed in the *pGlcT2* overexpressor line (Table 1), we assume that glucose released from the chloroplasts under long-day or continuous light conditions is phosphorylated by the cytosolic hexokinase and apparently drained into sucrose biosynthesis, leading to increased sucrose levels (Fig. 7*C*).

The clear, more than twofold, increase of starch in *pGlcT2* overexpressor plants (Fig. 7*D*) is fully in line with the strongly increased expression of the *GPT2* gene (5.2 log2-fold) coding for the chloroplast located glucose-6-phosphate (Glc6P) importer (Table 1). It is known that *GPT2* expression is positively affected by glucose (48) and in feeding experiments we showed that glucose provision to the cytosol leads not only to increased Glc6P transport activity in isolated chloroplasts but also to high rates of Glc6P-dependent starch synthesis in isolated chloroplasts (71). Thus, the latter experimental treatment (71) mimics the metabolic changes in *pGlcT2* overexpressors under long-day or continuous light conditions, with a consequence of these molecular and biochemical reactions being an accordingly impaired carbohydrate metabolism (Fig. 7, *A*–*D*).

Interestingly, the observation that increased sugar export activity out of an organelle into the cytosol leads to a subsequent stimulation of another sugar import system in the same organelle has also been made on vacuoles. In this organelle, an increased sugar export by overexpression of the glucose transporter ERDL6 provokes a stimulated vacuolar sucrose import mediated by the glucose-induced Tonoplast Sugar Transporter2 (29). This simultaneous process leads in sum, despite glucose export out of the vacuole being stimulated, to even higher total leaf sugar levels (29).

The decreased growth efficiency of *pGlcT2* overexpressor mutants under long-day and, most pronounced, under continuous light conditions, are fully explainable by altered gene expression and impaired photosynthetic properties. All genes monitored coding for proteins of photosystem I (PS_I_) or PS_II_, and even for Calvin cycle enzymes are down regulated in *pGlcT2* overexpressor plants, reaching a value of up to −4.23 log2 fold (Table 2). Simultaneously, the quantum yield of overexpressor plants is reduced, while the dissipation of excess light energy *via* non-photochemical quenching (NPQ) is increased (Fig. 8, *C* and *D*). In fact, the down regulation of so many PS_I_ and PS_II_, and Calvin cycle enzymes is a further indicator for cytosolic glucose accumulation in *pGlcT2* overexpressors, because the expression of wide number of photosynthesis-related genes is decreased by glucose (46).

Anyhow, *pGlcT2* overexpressor- and knockout mutants showed opposite growth patterns and individual carbohydrate levels during early seedling development (Fig. 5, *A* and *B*). In contrast, overexpressor mutants and knock-out plants exhibit in tendency similar phenotypes and similar carbohydrate levels during growth under extended light periods (Figs. 6 and 7). Latter observations are unexpected because in extended light periods both types of genetic modifications lead to individual gene expression patterns (Table 1). However, it has frequently been observed that impaired growth of plant mutants provokes the accumulation of various carbohydrates (63, 72, 73) which might explain this coincidence.

All in all, we showed that pGlcT2 is a chloroplast-located glucose transporter that’s activity has to be controlled to prevent developmental defects during seedling development and to allow proper acclimation to extended light periods. Taking pGlcT2 as an example we provided evidence that a sugar transporter, catalyzing facilitated diffusion, can alter the direction of net transport in dependence upon the developmental status and the growth conditions of Arabidopsis.

## Experimental procedures

### Alignment of pGlcT2 with other sugar transporters and phylogenetic analysis of the distribution pGlcT2 genes in plant species

Alignment of pGlcT2 with other sugar transporters (VGT1: *At3g03090*, pSUT: *At5g59250*, and pGLCT: *At5g16150*) was done using MEGA11 software (74) based on ClustalW (75) alignment of the corresponding amino acid sequences. The alignment was visualized using GeneDoc (76). The putative chloroplast transit peptides of the different transporters were predicted using TargetP (77) based on their amino acid sequence. Transmembrane regions of the pGlcT2 protein were predicted using DeepTMHMM (78).

Using pGlcT from *A. thaliana* as a query, 113 protein sequences with a BLAST E-value below 10^-85^ were selected from a broad phylogenetic range of 28 fully sequenced plant species whose genomes are deposited in the Phytozome database. N-terminal sequences of little conservation were removed (Data S1), the sequences aligned with MUSCLE at the European Bioinformatics Institute website, and a maximum likelihood tree was constructed with the MEGA software package (Tamura *et al*., 2021). The Neighbor-Join and BioNJ algorithms and the JTT model were used. A discrete Gamma distribution with five categories was assumed (+G, parameter = 1.2039) while 7.17% sites were set to be invariable. All positions with less than 95% site coverage were eliminated resulting in 446 positions in the final dataset. Affinity Designer software (V1.10) was used for the graphical representation.

### *E. coli* strains and bacterial growth conditions

*E. coli* XL1-Blue Mrf’ Kan (Stratagene, San Diego, CA, USA) was used for cloning. Strains were grown aerobically at 37 °C on LB medium (1% (w/v) tryptone, 1% (w/v) NaCl, 0.5% (w/v) yeast extract), containing 25 μg/ml chloramphenicol when they contained pABS-family plasmids.

### Genetic methods

The coding sequence without stop codon of *At1g05030* (= *pGlcT2*) was amplified from leaf cDNA with primers P1136 (5′-TTA TCG ATA AAA TGT GGG TGA CGA AT-3′) and P1137 (5′-TTC CCG GGA CTC AGG TCG TCT C-3′), introducing a *ClaI* and a *XmaI* site, respectively. The amplified fragment was cloned into pJet1.2 (Thermo-Fischer, Jena, Germany), generating construct H437. The full-length coding region of *pGlcT2* was amplified using the primers pGlcT2a-F-NdeI (5′-TGA ATC ATA TGG TGA CGA ATA CCG TAC TTC TAT AT-3′) and pGlcT2-R-*BamHI*(5‘-ACC AAG GAT CCA CTC AGG TCG TCT CTG GAG TTC-3'), using construct H437 as a template, and cloned into pABS-*tatABC*-H6 (79) using the *NdeI*/*BamHI* restriction site, resulting in pABS-*pGlcT2a*-H6. Note that we omitted Trp-2 from the sequence, as a possible removal of the N-terminal N-formyl-methionine would have generated a highly unstable protein (80). As the N-terminal plastid-targeting transit peptide, which mediates protein targeting across the plastid envelope membranes, is not required and potentially unwanted for experiments in *E. coli*, we also constructed a plasmid for the expression of a variant of the *pGlcT2* gene that lacks the coding region for this transit peptide. The transit peptide was predicted based on TargetP 2.0 (77) and alignments with related transporters. A synechococcal putative major facilitator superfamily sugar transporter (GenBank accession MBF2076211.1) was most similar (ca. 34% identity, 83% query cover), the alignment starting with Val-48, suggesting that the transit peptide should be the N-terminal region up to around Val-48. TargetP 2.0 predicts a more likely cleavage site in three residues distance (Leu-51). We chose to place the N-terminal Met before Val-48 which should permit membrane targeting in any case. To construct the gene for this “mature” transit peptide-lacking transporter, the forward primer pGlcT2b-F-NdeI (5’-TGA ATC ATA TGG TTA CGA CAT TGT CGA CGA AGA AAC C-3′) was used in combination with the primer *pGlcT2*-R-*BamHI*, and *NdeI*/*BamHI* cloning resulted in the plasmid pABS-*pGlcT2b*-H6. For controls, we used the plasmid pABS-*tatC* for the production of TatC (81). TatC is an unrelated membrane protein that is involved in protein translocation (40).

### Complementation analyses

The functionality of pGlcT2 was initially examined by complementation of the glucose-uptake defect in *E. coli* MG1655 Δ*ptsG* Δ*manXYZ* (41). MG1655 Δ*ptsG* Δ*manXYZ* per se does not grow on glucose as only a carbon source, and if the expression of *pGlcT2* enables growth, this indicates functionality in glucose uptake. For this complementation analysis, MG1655 Δ*ptsG* Δ*manXYZ* was transformed with pABS-*pGlcT2a*-H6, pABS-*pGlcT2b*-H6, or pABS-*tatC* (negative control). Cultures in M9 medium with 0.4% glucose as the only carbon source was started with an optical density measured at 600 nm (OD600) of 0.02 using over-night cultures of the strains that had been grown in M9 medium with 0.4% xylose as the only carbon source. Strains were grown at 37 °C in shaking 96-well plates (culture volume: 200 μl) with technical triplicates, using measurements of the OD600 in 15 min intervals with the SpectraMax iD3 Microplate Reader (Molecular Devices, San Jose, CA, USA).

### Transport studies using ^14^C-labeled glucose

For radioactive uptake studies overnight cultures of *E. coli* MG1655 Δ*ptsG* Δ*manXYZ* expressing either the functional sequence of *pGlcT2* or *TatC* were diluted with fresh M9-Xylose (0.4%)-medium to an OD600 of 0.2. After incubation and growth at 37 °C to an OD600 of ∼0.5, cells were harvested, washed with sterile water once and resuspended in uptake buffer (15 mM MES + 15 mM HEPES + 15 mM Tris + 5 mM MgCl2, pH 7.0) to an OD600 of 5. For time-dependent uptake studies, the reaction was started by the addition of a mixture of non-radioactive glucose and 1 μCi ^14^C-glucose, leading to a total concentration of 5 mM glucose in the reaction mixture. The reaction was incubated at 37 °C with steady agitation at 200 rpm for a total of 60 min. At the indicated timepoints (0, 15, 30, and 60 min) 100 μl of the reaction mixture was collected on filter paper (MCE Membrane Filter, 0.45 μm) and washed with a total of 5 ml of uptake buffer by vacuum filtration. The filter was transferred to scintillation vials containing 4 ml of Rotiszint eco plus (Carl Roth, Germany) and measured in a Tri-Carb 4810 TR scintillation counter (PerkinElmer, USA). For concentration-dependent uptake assay and determination of K_m_, uptake was performed as stated above with the alteration of induction of the uptake reaction by the addition of glucose leading to a final glucose concentration of 1, 2, 4, 8, and 16 mM in the reaction. Thereby the amount of radioactive glucose was unaltered at 1 μCi of ^14^C-glucose in each reaction. pH dependency was tested by performing uptake in uptake buffer buffered at the indicated pH of 5.0, 7.0 or 9.0 by addition of HCl or NaOH, respectively. Testing of pH dependency was done at an outer glucose concentration of 5 mM in the reaction mix. Transport specificity was tested by the addition of 10× excess concentration of competitive carbohydrates (glucose, fructose, sucrose, maltose, ribose, raffinose, and inositol). Given that the control uptake reaction was performed with an outer glucose concentration of 2 mM glucose containing 1 μCi ^14^C-glucose, competitive carbohydrates were added to a final concentration of 20 mM to the reaction mix. For analysis of concentration- and pH-dependency, as well as the transport specificity, the reaction was stopped after 20 min by collecting cells *via* vacuum filtration.

### Subcellular localization

To confirm the subcellular localization of pGlcT2, the Gateway entry vector pDONR/Zeo containing the coding sequence of *pGlcT2* was used to sub-clone the target sequence in the destination vector PUBDESTCgfp, a Gateway-compatible vector with a C-terminal GFP coding sequence under the control of the ubiqutin-10 gene promoter (PUBQ10) from Arabidopsis, *via* LR reaction. The Gateway forward (5ʹ-GGG GAC AAG TTT GTA CAA AAA AGC AGG CTT AAT GTG GGT GAC GAA TAC C-3ʹ) and reverse (5ʹ-GGG GAC CAC TTT GTA CAA GAA AGC TGG GTA ACT CAG GTC GTC TCT TGG-3ʹ) primers were used for the initial sub-cloning of the coding sequence of *pGlcT2* into the Gateway entry vector pDONR/Zeo. The corresponding destination vector was transiently expressed in leaf mesophyll cells of *Nicotiana benthamiana* to clarify the subcellular localization of the pGlcT2 protein according to Jung *et al.* (82). For analysis of the subcellular localization of pGlcT2-GTP fusion constructs a Leica TCS SP5II confocal laser scanning microscope (Leica biosystems, Wetzlar, Germany) was used and pictures were taken through the Leica HCX PL APO 63·/1.20 w mot CORR CS objective with a Vis Argon Laser with the settings 488 nm/495 to 520 nm suitable for GFP.

### Tissue localization and histochemical GUS analysis

To create constructs for investigation of pGlcT2 tissue distribution, a fragment of 1992 bp upstream of the *pGlcT2* start codon was amplified *via* PCR with specifically designed Gateway-attachment site overhangs for forward (5ʹ-GGG GAC AAG TTT GTA CAA AAA AGC AGG CTT AGA CGG ATT CCT ATA GCT GAC-3ʹ) and reverse (5ʹ-GGG GAC CAC TTT GTA CAA GAA AGC TGG GTA GAT CGG AGA GCT AGA CTA G-3ʹ) primers. After gel extraction and purification *via* the NucleoSpin Gel and PCR Clean-up kit (Machery Nagel, Düren, Germany), the desired fragment was sub-cloned to pDONR/Zeo and finally pMDC163 as entry and destination vector respectively. Latter vector finally expressing the GUS (b-GLUCURONIDASE) reporter gene under control of the pGlcT2 promotor region. The above-mentioned floral dip method was used for Agrobacterium-mediated transformation to Arabidopsis plants. Homozygous lines of transgenic ProGlcT2:GUS plants, were accessed by multiple screening of mutant lines on Hygromycin MS agar plates. Histochemical GUS staining was performed as conducted in An *et al*. (83). For this, transgenic ProGlcT2:GUS plants were cultivated under standard growth conditions. Plant tissues were stained by 5-bromo-4-chloro-3-indolyl-β-glucuronic acid (X-Gluc) solution according to Chardon *et al*. (2013). Tissue distribution of the ProGlcT2:GUS was monitored and imaged using a Leica MZ10F modular stereo microscope combined with a Leica DFC420 C digital microscope camera (Leica Biosystems, Wetzlar, Germany).

### Generation and preparation of mutants

To generate constructs for producing pGlcT2 overexpression lines, full length of coding sequence for *pGlcT2* (*At1g05030*) was amplified by specific designed Gateway primers (forward; 5ʹ-GGG GAC AAG TTT GTA CAA AAA AGC AGG CTT AAT GTG GGT GAC GAA TAC C-3ʹ and reverse; 5ʹ-GGG GAC CAC TTT GTA CAA GAA AGC TGG GTA TTA ACT CAG GTC GTC TCT G-3ʹ) *via* PCR followed by an extraction and purification from agarose gel using the NucleoSpin Gel and PCR Clean-up kit (Machery Nagel, Düren, Germany) following its user guidelines. The target fragment was sub-cloned through BP reaction into the Gateway entry vector pDONR/Zeo and then *via* LR reaction into the destination vector pK2GW7 containing a 35S-CaMV promotor to create overexpress lines. The Agrobacterium-mediated transformation by simplified floral dip method was used to generate stable transformed overexpression plants (Clough and Bent, 1998). qRT-PCR was performed in order to select the two independent overexpression lines (*GlcT2#8* and *GlcT2#5*). Corresponding homozygote lines were produced by multiple screening on MS agar plates *via* BASTA resistance system. Seeds of the T-DNA insertion line (*glct2* [SALK_052078]) were provided from the Nottingham Arabidopsis Stock Centre, NASC (University of Nottingham, UK) For complementation of the knockout line, the expression construct produced for the generation of the overexpression lines was transformed to the background of knockout plants by Agrobacterium-mediated transformation method *via* floral dip as described above. qRT-PCR was conducted in order to select a complementary line (*glct2-comp*).

### Plant material and experimental conditions

Wild-type seeds of *A. thaliana* (Col-0) and corresponding mutants including the two overexpression lines (*GlcT2#8* and *GlcT2#5*), knockout (*glct2* [SALK_052078]) and the complementary line (*glct2-comp*) were cultivated under different experimental conditions. For soil experiments, seeds were sown on standard soil (ED-73; Einheitserde Patzer; Sinntal-Altengronau, Germany), stratified at 4 °C/darkness for 48h followed by transfer to short-day (10h light/14h darkness) growth chambers (22 °C and a light intensity of 120 μmol quanta m^−2^ s^−1^). To investigate the effects of different light periods on plant performance, 1-week-old seedlings were transferred to either short-day (standard condition) or long-day conditions (16 h light and 8 h darkness). To apply an artificial continuous light condition, 24 days old plants grown under short day were transferred to a specific growth chamber (percival) with a continuous light intensity of 100 to 120 μmol quanta m^−2^ s^−1^ for 10 to 12 days. To monitor the effect of the absence or presence of sugars on the mutant’s growth, the agar plates containing half strength of MS salts, 0.05% (w/v) MES, 0 to 0.5% sucrose and 0.8% agar (adjust to PH 5.7 with KOH) were used. To analyze primary root length, ImageJ 1.46v software was utilized. For RNA and sugar extractions, plant tissues were harvested at desired time points and frozen immediately in liquid nitrogen. All samples were kept in −80˚C until analysis.

### cDNA synthesis and gene expression analyses

RNA was extracted from 50 mg of frozen powdered tissues by the NucleoSpin RNA Plant Kit (Macherey-Nagel), based on the manufacturer’s protocol. The qScript cDNA Synthesis Kit (Quantabio) was used for cDNA synthesis. The list of primers used for gene expression analysis by qRT-PCR was presented in Table S1. To normalize transcript levels of target genes, the protein phosphatase 2A (PP2AA3; *At1g13320*) and the SAND family protein (*At2g28390*) were used as reference genes. The relative gene expression was calculated by using the 2-ΔΔCT formula (84).

### Carbohydrate extraction and quantification

Ground fine tissues (50 mg) were used to extract total sugars by adding 500 μl of 80% absolute ethanol at 80 °C while shaking on a thermo-block for 30 min. The homogenized mixture was centrifugated at 16,000*g* (10 min at 4 °C), the supernatant was removed for evaporation by a vacufuge concentrator and finally, the dried pellet dissolved in 500 μl of dd H_2_O to use for sugar quantification. Starch hydrolysis was applied on the pellet separated after removing the ethanolic extract. For that, the pellet was washed twice with 80% Ethanol and once with dd H_2_O. To inactivate endogenous hydrolysis enzymes and sterilization, 200 μl of dd H_2_O was added to the washed pellet, and the samples were autoclaved at 120 °C for 20 min. After cooling down, 200 μl of master mix (45 μl/ml amyloglucosidase and 1 mg/ml α-amylase dissolved in 200 mM sodium acetate; pH = 4.8) was added to each sample, remained at 37 °C overnight, and ultimately inactivated the enzymatic reaction by keeping samples at 95 °C for 8 to 10 min. Final centrifugation was applied on cooled samples (16,000*g* for 10 min at room temperature). The supernatant was used for sugar quantification. To quantify the sugars (glucose, fructose, sucrose), the pellet produced after evaporation of ethanol was dissolved in and used for sugar determination by an NADP-coupled enzymatic test (85).

### Photosynthetic activity

The efficiency of photosynthesis was investigated in mutants grown under short-day and continuous light for 3 weeks. For this, the imaging-PAM M-Series-System (Walz) was utilized to determine photosynthetic plant performance by evaluation of plant response to different light intensities (light curve). To achieve this, plants were adapted to the darkness for 15 min prior to the exposure to different light intensities which were started from 1 PAR (μmol photons m-2 s-1) and ended up at 726 PAR. The quantum yield of non-photochemical quenching [Y(NPQ)] and the effective quantum yield of PSII [Y(II)] were calculated as described previously (4).

### RNA seq analysis

RNA-Seq analysis was conducted on the wild-type and mutant plants exposed to continuous light conditions. For this, plants exposed to the continuous light were harvested in four biological replicates and immediately frozen at −80 °C. The frozen ground tissues were used for RNA extraction and the quality of RNA was determined using the NanoPhotometer N50 (Implen, München, Germany). The RNA samples were transported on dry ice to Novogene UK for subsequent RNA-Sequencing and analysis. From the provided RNA-Seq data genes of interest were extracted and log2 fold-change ratios between wild types and mutants were calculated.

## Data availability

All data supporting the findings of this study are available within the paper and within its supporting information published online. RNASeq data is made available on GEO repository and can be accessed at https://www.ncbi.nlm.nih.gov/geo/query/acc.cgi?acc=GSE223330.

## Supporting information

This article contains supporting information citing following publications (34, 74, 75, 76, 77, 78). Supplementary Table S1, Supplementary Figures S1–S4, and Supplementary Data S1.

## Conflict of interest

The authors declare that they have no conflicts of interest with the contents of this article.
