# Supplementary material for: The novel chloroplast glucose transporter pGlcT2 affects adaptation to extended light periods
Source: J Biol Chem. 2023 Apr 23;299(6):104741. doi: 10.1016/j.jbc.2023.104741 (PMC10318453; doi:10.1016/j.jbc.2023.104741)
Supplement: Supporting information [file mmc1.docx]

**Supporting Information**

**The novel chloroplast glucose transporter pGlcT2 affects adaptation to extended light periods**

Marzieh Valifard, Alisdair R. Fernie, Anastasia Kitashova, Thomas Nägele, Rebekka Schröder, Melissa Meinert, Benjamin Pommerrenig, Denise Mehner-Breitfeld, Claus-Peter Witte, Thomas Brüser, Isabel Keller and H. Ekkehard Neuhaus

**Supporting Table 1.** List of primers used in this study

**Supporting Figure S1.** Alignment of *Arabidopsis thaliana* VGT1, pSuT, pGlcT and pGlcT2.

**Supporting Figure S2.** Sequence clades of plant pGlcT, pGlcT2 and SGB1 transporters

**Supporting Figure S3.** Characterization of *Arabidopsis thaliana pGlcT2* mutant lines

**Supporting Figure S4.** Growth analysis of *Arabidopsis thalina pGlcT2* mutant lines short day control

**Supporting Table S1:** List, purpose and sequence of the primers used in this study.

| **Purpose** | **Accession number** | **Primer name** | **Sequence** |
| --- | --- | --- | --- |
| gene expression (qRT-PCR) | AT2G28390 | SAND family-FW | 5ʹ-AACTCTATGCAGCATTTGATCCACT-3ʹ |
|  |  | SAND family-RE | 5ʹ-TGATTGCATATCTTTATCGCCATC-3ʹ |
|  | AT1G13320 | PP2A subunit PDF2-FW | 5ʹ-TAACGTGGCCAAAATGATGC-3ʹ |
|  |  | PP2A subunit PDF2-RE | 5ʹ-GTTCTCCACAACCGCTTGGT-3ʹ |
|  | AT4G15920 | SWEET17-FW | 5ʹ-AGTGACAACAAAGAGCGTGAAATAC-3ʹ |
|  |  | SWEET17-RE | 5ʹ-ACTTAAACCGTTGCTTAAACCACCC-3ʹ |
|  | AT1G05030 | pGlcT2-FW | 5ʹ-ACGAGTGGTGCTCAAGCAAGTC-3ʹ |
|  |  | pGlcT2-RE | 5ʹ-TAAGACGCACACAGAGCACCTG-3ʹ |
|  | At1G75220 | ERDL6-FW | 5ʹ-GGTCGTCGGCTTCTGCTTACTATC-3ʹ |
|  |  | ERDL6-RE | 5ʹ-GCCTTGTAATTGTTGCAGCTGCT-3ʹ |
|  | At3G16690 | SWEET16-FW | 5ʹ-GAGATGCAAACTCGCGTTCTAGT-3ʹ |
|  |  | SWEET16-RE | 5ʹ-GCACACTTCTCGTCGTCACA-3ʹ |
|  | At1G29930 | CAB1-FW | 5ʹ-TTACTTGCGCCACACTCTCACC-3ʹ |
|  |  | CAB1-RE | 5ʹ-TTTCCGGTCAAAGCAGGAGAGG-3ʹ |
|  | At1G15500 | NTT2-FW | 5ʹ-ATGGAAGGTCTGATTCAAACC-3ʹ |
|  |  | NTT2-RE | 5ʹ-TAAATGCCAGTAGGAGTAGATTTCTC-3ʹ |
|  | AT1G61800 | GPT2-FW | 5´-TCTATTGCCGTGGAAGGTCCT-3 |
|  |  | GPT2-RE | 5´-ACTTTGTGCCACTACCCACCA-3 |
| homoz. testing | AT1G05030 | pGlcT2-LP | 5ʹ-CACTCAAGCTTCCGTCGTTAG-3ʹ |
|  |  | pGlcT2-RP | 5ʹ-GAGGAAAAGCCGATAACCATC-3ʹ |
|  |  | LB | 5ʹ-ATTTTGCCGATTTCGGAAC-3ʹ |
| semiquant. PCR | AT1G05030 | pGlcT2-full_length-FW | 5ʹ-ATGTGGGTGACGAATACC-3ʹ |
|  |  | pGlcT2-full_length-RE | 5ʹ-TTAACTCAGGTCGTCTCTG-3ʹ |
|  | AT1G07940 | EF1a-FW | 5´-GAGACCACCAAGTACTACTGCAC-3´ |
|  |  | EF1a-RE | 5´-GTTGGTCCCTTGTACCAGTCAAG-3´ |


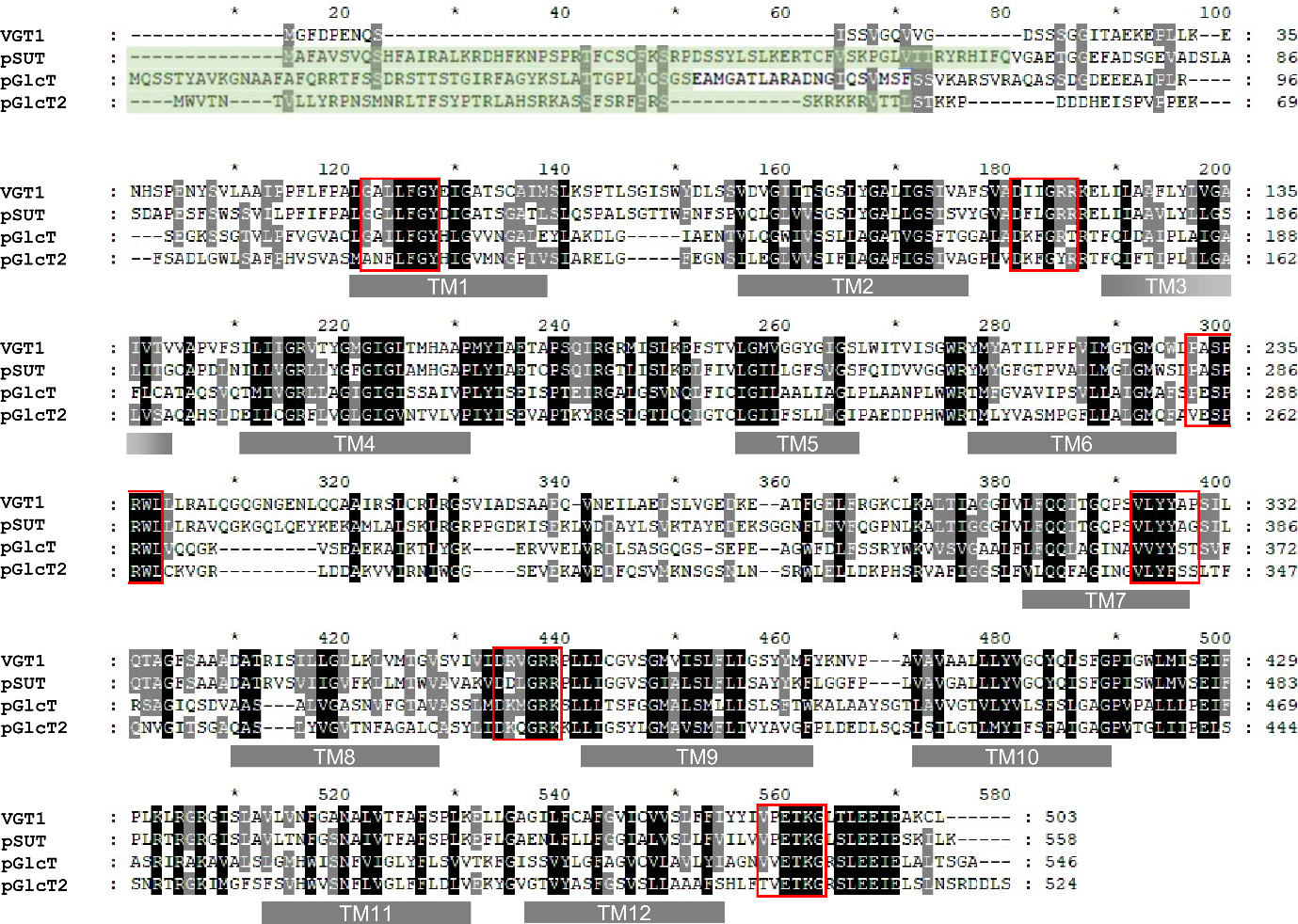


**Supporting Figure S1. Alignment of *Arabidopsis thaliana* VGT1, pSuT, pGlcT and pGlcT2.**

Comparative amino acid sequence alignment was done using MEGA11 software (74) and calculated using ClustalW (75). Alignment was visualized using GeneDoc (76). Conserved amino acids are shaded in black (100% AA identity in all sequences) or dark grey (80% AA identity in all sequences). Prediction of the putative chloroplast transit peptide (shaded in green) was done using TargetP (77). Conserved sugar transporter motifs are marked in red (34). Transmembrane regions of pGlcT2 were predicted using DeepTMHMM (78) and are visualized as grey boxes (TM1- TM12).


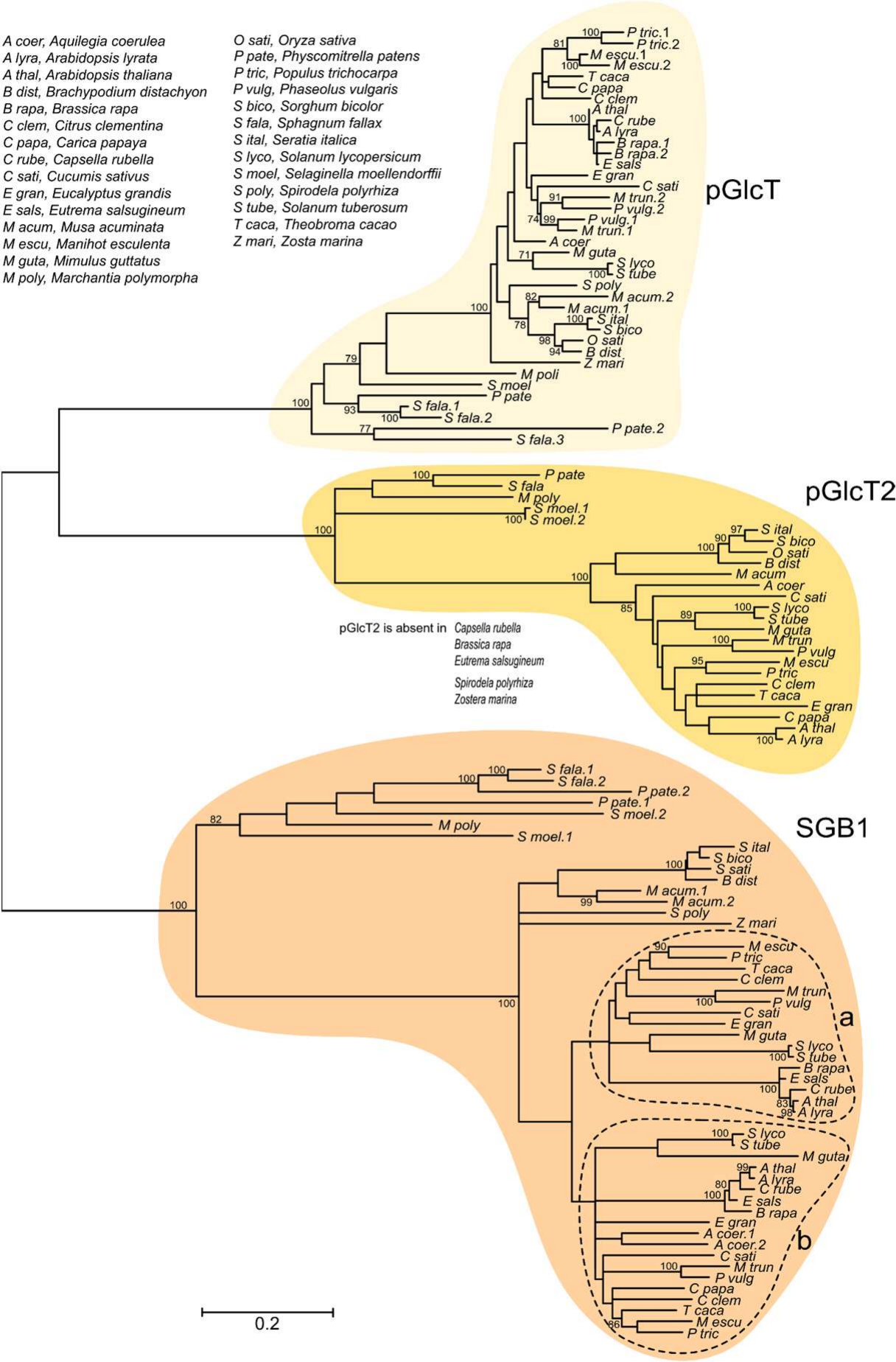


**Supporting Figure S2. Sequence clades of plant pGlcT, pGlcT2 and SGB1 transporters indicating functional groups.**

The shown maximum likelihood tree was constructed with the MEGA software package (74) using a multiple alignment of pGlcT and the closely related pGlcT2 and SGB1 proteins from a broad phylogenetic range of 28 fully sequenced plant species. The tree with the highest log likelihood is displayed. 1000 bootstraps were performed, and bootstrap values are only shown at branches with higher than 70% bootstrap support. Branch lengths represent the number of substitutions per site. Main clades separated with 100% support at the base of the tree comprise the pGlcT transporters and the SGB1 transporters, which are both represented in all analyzed plants, as well as the pGlcT2 transporters, which are absent in five of the 28 species – in particular several Brassicaceae species lack pGlcT2. Dicotyledons generally possess two SGB1 proteins with only subtle sequence differences which did not result in a statistically sound phylogenetic separation into clades. Nonetheless, they were labeled SGB1a and SGB1b (surrounded by dashed lines) because they can be discerned based on a few indicative amino acids.


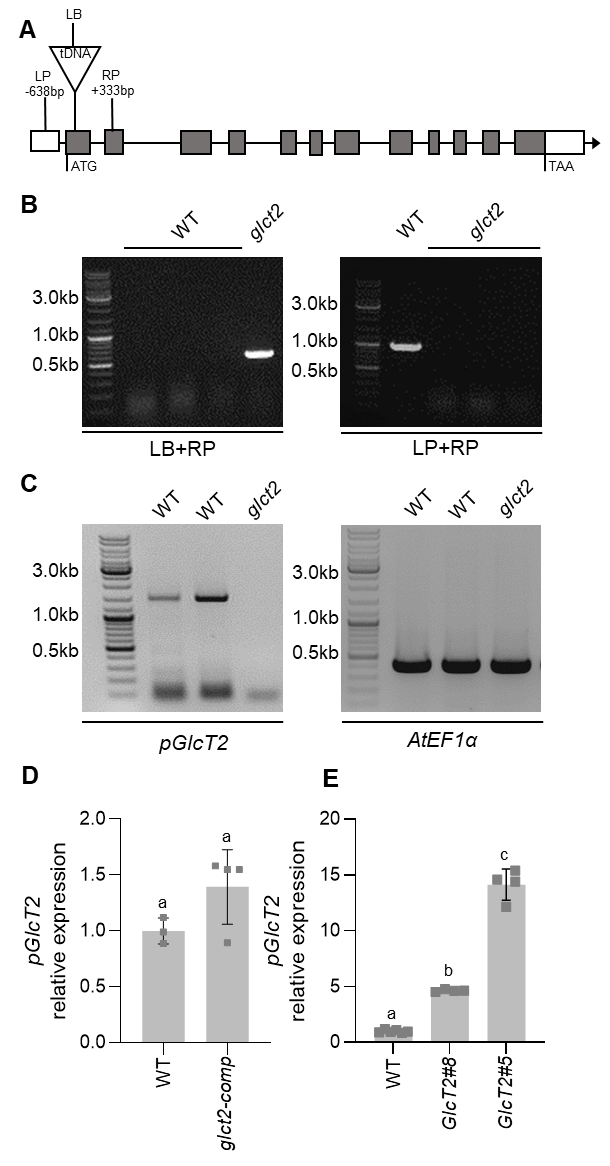


**Supporting Figure S3. Characterization of *Arabidopsis thaliana* pGlcT2 mutant lines**.

Schematic representation of the position of tDNA-insertion in the SALK_052078 mutant line, further referred to as *glct2* (A), as well as homozygosity screening of the mentioned mutant using LB and RP primers resulting in a product size of 431-731bp in the *glct2* line and using LP and RP primers resulting in an amplification product of 972 bp in wild types (B). Semi-quantitative expression analysis using *pGlcT2* full-length amplification primers (1574bp) and *AtEF1α* reference primers (400bp) performed on cDNA using 28 amplification cycles revealed absence of *pGlcT2* expression in *glct2* lines (C). Note different *pGlcT2* signal intensity in wild types due to natural variation (C). *pGlcT2* expression level in the complementation line *glct2-comp* (D) and the two overexpressor lines *GlcT2#5* and *GlcT2#8* was analyzed using qRT-PCR.


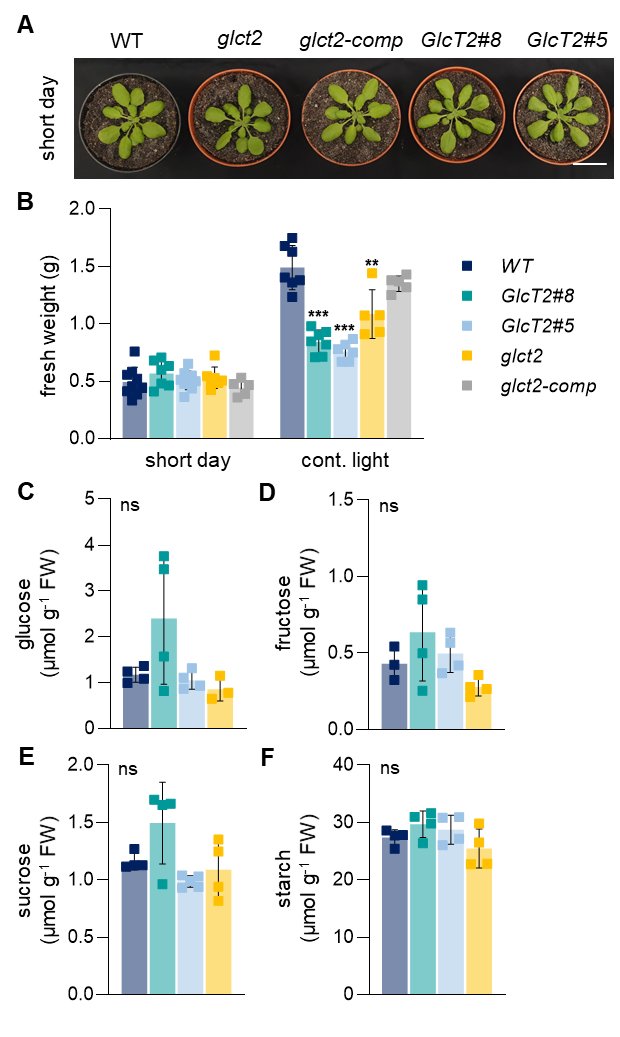


**Supporting Figure S4. Growth analysis of *Arabidopsis thaliana* pGlcT2 mutant lines under short day conditions to the corresponding analysis under continuous light conditions.**

Plant phenotype (A), plant biomass (B), and glucose content (C), fructose content (D), sucrose content (E), and starch content (F) of plants grown under short day conditions. Seeds were sown on soil, stratified at 4°C/darkness for 48 hours, and then grown under short day conditions for 24 days and another 10-12 days under either short day (control) or continuous light conditions. Control plants shown here serve as same-age control for plants treated with continuous light (see Figure 6 and Figure 7) to minimize effects on phenotype and metabolite levels due to a difference in developmental age of plants. Plants were harvested at midday and tissues were frozen in liquid nitrogen and stored at -80 °C until analysis. Scale bar represents 2 cm in (A). Results are means of 5-7 replicates ± SD. Significant differences were calculated between WT and corresponding mutants within one condition using Student´s t-test detecting no significant differences.
